# Coevolution of female and male genital components to avoid genital size mismatches in sexually dimorphic spiders

**DOI:** 10.1186/s12862-016-0734-9

**Published:** 2016-08-17

**Authors:** Nik Lupše, Ren-Chung Cheng, Matjaž Kuntner

**Affiliations:** 1 Institute of Biology, Research Centre of the Slovenian Academy of Sciences and Arts, Ljubljana, Slovenia; 2 Department of Entomology, National Museum of Natural History, Smithsonian Institution, Washington, DC 20013-7012 USA

**Keywords:** Sexual size dimorphism, Sexual genital size dimorphism, External genitalia, Internal genitalia, Intromittent genitalia, Non-intromittent genitalia, Sexual selection

## Abstract

**Background:**

In most animal groups, it is unclear how body size variation relates to genital size differences between the sexes. While most morphological features tend to scale with total somatic size, this does not necessarily hold for genitalia because divergent evolution in somatic size between the sexes would cause genital size mismatches. Theory predicts that the interplay of female-biased sexual size dimorphism (SSD) and sexual genital size dimorphism (SGD) should adhere to the ‘positive genital divergence’, the ‘constant genital divergence’, or the ‘negative genital divergence’ model, but these models remain largely untested. We test their validity in the spider family Nephilidae known for the highest degrees of SSD among terrestrial animals.

**Results:**

Through comparative analyses of sex-specific somatic and genital sizes, we first demonstrate that 99 of the 351 pairs of traits are phylogenetically correlated. Through factor analyses we then group these traits for MCMCglmm analyses that test broader correlation patterns, and these reveal significant correlations in 10 out of the 36 pairwise comparisons. Both types of analyses agree that female somatic and internal genital sizes evolve independently. While sizes of non-intromittent male genital parts coevolve with male body size, the size of the intromittent male genital parts is independent of the male somatic size. Instead, male intromittent genital size coevolves with female (external and, in part, internal) genital size. All analyses also agree that SGD and SSD evolve independently.

**Conclusions:**

Internal dimensions of female genitalia evolve independently of female body size in nephilid spiders, and similarly, male intromittent genital size evolves independently of the male body size. The size of the male intromittent organ (the embolus) and the sizes of female internal and external genital components thus seem to respond to selection against genital size mismatches. In accord with these interpretations, we reject the validity of the existing theoretical models of genital and somatic size dimorphism in spiders.

**Electronic supplementary material:**

The online version of this article (doi:10.1186/s12862-016-0734-9) contains supplementary material, which is available to authorized users.

## Background

Traits that arise through sexual selection result in benefits from higher reproductive success [1]. While sex-specific armaments and genital morphologies may be clear-cut examples of traits that respond to sexual selection [2–4], it remains unclear how sex-specific body size variation relates to genital size differences between the sexes. Limited research in this area has only examined the patterns of intraspecific scaling of genital traits [5–9], but comparative research is lagging behind. While most morphological features tend to increase in size along with total somatic size, this does not necessarily hold for genitalia because divergent evolution in somatic size between the sexes would cause genital size mismatches [10]. Despite the importance of somatic and genital size evolution for our understanding of sexual selection, the patterns remain largely unexplored.

To investigate this interplay, studies on sexually dimorphic organisms need to focus on the interaction between sexual size dimorphism (SSD), and sexual genital size dimorphism (SGD). However, SSD, an evolutionary phenomenon where the sexes exhibit considerable size differences [11–16], is much better understood than SGD. Furthermore, while research on clades with male-biased SSD may have converged on its causes and consequences that relate to sexual selection [17–20], in clades that exhibit a strong female-biased SSD, such as many invertebrates, size evolution responds to a mix of sexual and natural selection [13, 21–25].

Spiders may be among the most promising model groups for addressing this topic, as they show a wide range of SSD from monomorphic clades to clades with moderately male-biased and to extremely female-biased taxa [13, 22, 26, 27]. Repeated evolution of extremely female-biased SSD in spiders suggests strong fecundity selection on females [28, 29] and sexual selection [10] on males [13, 30]. SSD also skews sex ratios to make them male biased. Consequently, polyandry leads to shared paternity [31, 32], and resulting sperm competition drives the evolution of male genital morphologies and mating behaviours that function to monopolize females [33]. Examples are shifts in male genital complexity that facilitate genital mutilation and plugging [29, 33]. Because such male adaptations necessarily affect female mating rates, they are viewed to be sexually conflicted [29, 34]. Females respond with counter adaptations in likely bursts of evolutionary arms race, but the study that hypothesized this [29] only recognized counter shifts in female genital complexity, while genital size evolution in both sexes remains unexplored.

**Fig. 1:**
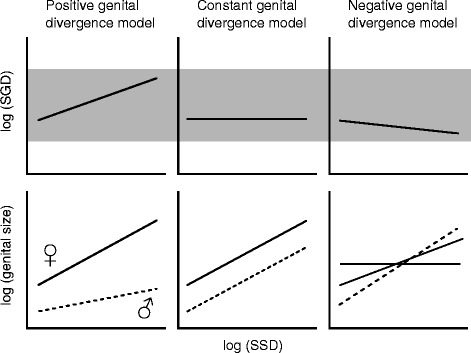
Theoretical relationships between sexual genital size dimorphism (SGD) and female-biased sexual size dimorphism (SSD). The upper figures are the expected relationships of SGD to SSD, the lower figures are the predicted patterns of female (*solid line*) and male (*dashed line*) genital size on SSD. *Shaded area* indicates theoretical fits of genital sizes. Values that fall out of this area would imply genital size mismatch

Ramos et al. [10] examined the correlation between a female-biased SSD and SGD in orb-weaving spiders, and allowed for three theoretical models (Fig. 1): (1) The “positive genital divergence” model suggests a positive correlation between SGD and SSD (Fig. 1). This would result in much larger female genitalia due to faster rates of growth through evolutionary time, consequently disturbing the SGD ratio between the sexes, until halted by selection to avoid complete genital size mismatch. (2) The “constant genital divergence” model suggests that SGD remains constant as SSD increases (Fig. 1). Natural selection should accordingly favour a constant ratio of female to male genital size as body sizes diverge [35]. Thus, in order to avoid genital size mismatches, male and female genital sizes would show comparable correlations with change in SSD. This model would be consistent with the “one size fits all” hypothesis [6, 36]. (3) The “negative genital divergence” model that has so far not been documented, predicts that as SSD increases, SGD decreases (Fig. 1). This scenario would imply a positive correlation between SSD and male genital size, while female genital size would either stagnate, or slightly increase with SSD (Fig. 1).

Whether or not any of these three models explain SSD and SGD evolution in other invertebrates is unknown, but the study on spiders [10] supported the positive genital divergence model. That study was performed at a high taxonomic level using sporadic araneoid exemplars. Here, we provide comparative tests of the relationships between SSD and SGD at the species level in the family Nephilidae, using a phylogeny with branch length information, and detailed measures of male and female somatic and genital sizes. We first test whether macroevolutionary changes in genital size relate to the changes in body size in each sex. Nephilid (and other entelegyne) spiders have both external and internal female genital structures that are also functionally distinct. The external genital parts interact with male genitalia, and the internal ones lead sperm to its storage, the spermathecae, then direct it towards fertilization in *uterus externus* [37]. Similarly, paired male secondary genitalia (pedipalps) can be divided into non-intromittent parts (the bulb containing a sperm reservoir, with associated sclerites and membranes), and the intromittent part, the embolus [37]. Therefore we obtained separate size measures for components of external and internal genital parts, and for non-intromittent and intromittent components, and then evaluated SGD (size of female/male genitalia) based on any combination of the metrics. Because of their functional differences, it may be plausible that external and internal genital sizes, as well as non-intromittent and intromittent genital sizes, may respond differently to selection pressures. If so, some but not all genital components could evolve to avoid genital size mismatches that would otherwise arise as consequences of SSD. We also predicted to find no correlation between SSD and SGD, which might in the case of constant SGD values lend support to the “constant genital dimorphism” model, and in the case of variable SGD values suggest phylogenetic decoupling.

## Methods

### Taxon sampling

Nephilids are models in SSD research [26, 28, 37–40]; their independent size evolution in females and males has been described as “sexually dimorphic gigantism” [13]. However, their coevolutionary patterns of somatic and genital sizes, and of SSD and SGD, have remained unexplored.

We examined the correlation between body and genital size evolution in 14 selected nephilid species that differ in levels of SSD and represent all genus-level clades on nephilid phylogeny (Additional file 1: Figure S1). In total, we measured (detailed below) 126 individuals, 62 of them males and 64 females (doi:10.5061/dryad.m0hd2). The genus *Nephilengys* was represented by *N. malabarensis* (N females =5 ; N males =5) and *N. papuana* (4;5), the genus *Herennia* by *H. etruscilla* (5;3) and *H. multipuncta* (5;5), the genus *Nephilingis* by *N. cruentata* (5;5) and *N. livida* (5;5), the genus *Clitaetra* by *C. episinoides* (4;3) and *C. irenae* (5;5), the clade with type *Nephila* was represented by *N. pilipes* (5;5) and *N. constricta* (4;4), and the clade that contains all other species currently assigned to *Nephila* (labeled as “Nephila” in Additional file 1: Figure S1; for details, see [40]), was represented by *N. clavata* (4;4), *N. clavipes* (4;5), *N. fenestrata* (4;3), and *N. komaci* (5;5).

### Size measurements

We performed size measurements of both somatic and genital features using distances between easily recognizable landmarks on rigid structures. Because only adult orb-weavers possess genitalia, we only measured sizes in adult spiders. Likewise, studies on SSD in spiders exclusively measured adults [26, 29, 41]. In nephilids, male genitalia (modified pedipalps) are conspicuous paired structures whose landmarks are straightforward to define and homologize [37]. On the other hand, the female external (also epigynal) genital area in nephilids is often inconspicuous, and more challenging to precisely define. Similarly, it was difficult to locate precise landmarks in internal genital ducts of females [37]. Thus, for precise, repeatable measurements we dissected the genitalia and aligned the structures in consistent orientations [6].

**Fig. 2:**
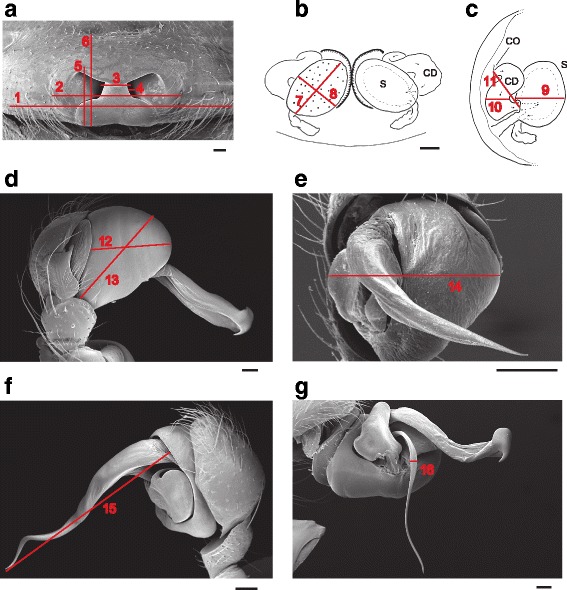
Measured genital parameters. **a** female epigynum, ventral view (*Nephilengys malabarensis*); **b**-**c** female internal genital tract (*Herennia multipuncta*) in dorsal (**b**) and ectal view (**c**); **d** male palp, ectal view (*Nephila fenestrata*); **e** male palp, apical view (*Clitaetra clathrata*); **f** male palp, mesal view (*Nephila constricta*); **g** expanded and rotated male palp (*Nephila fenestrata*). All scales = 0.1 mm. Abbreviations: 1 = Reproductive area width; 2 = Epigynal area width; 3 = Distance between copulatory openings; 4 = Copulatory opening width; 5 = Epigynal area length; 6 = Reproductive area length; 7 = Spermatheca height; 8 = Spermatheca width; 9 = Spermatheca length; 10 = Copulatory duct width; 11 = Copulatory duct length; 12 = Pedipalp bulb height; 13 = Pedipalp bulb length; 14 = Pedipalp bulb width; 15 = Embolic conductor length; 16 = Embolus width; CD = Copulatory duct, CO = Copulatory opening; S = Spermatheca

We measured four somatic characters [12]: body length, first leg tibia + patella length, carapace width, carapace length; and sixteen genital characters [37, 42]: males - pedipalp bulb length, height and width, embolic conductor length, and embolus width, the latter (and the derived embolus volume) representing the intromittent part of the palp and the former all being part of the non-intromittent secondary male genitalia (Fig. 2). In females, the internal genital measures were spermathecal length, height and width, and copulatory duct length and width, and the external genital measures were copulatory opening width, distance between the copulatory openings, epigynal area length and width, and reproductive area length and width (Fig. 2).

For detailed examination of female internal genital structures we dissected the genital area, cleared it from surrounding tissues, then macerated it in 5 % KOH [8] before gently soaking it in water, and microscopically examining the revealed structures in 70 % EtOH. All measurements (doi:10.5061/dryad.m0hd2) were performed using the Leica Application Suite software (Leica, Bannockburn, IL).

### Mathematical transformations

Prior to the implementation of phylogenetically independent contrasts in PDAP package of Mesquite [43], we square-root transformed female epigynal and reproductive area. We used cube-root transformation for male pedipalp, pedipalp bulb and embolus volume and for female spermathecal volume. These transformations enabled us to proceed with correlation analyses as both somatic and genital variables were brought to comparable biological scales [10].

The respective volumes of pedipalp bulb, embolus, pedipalp and spermatheca were calculated from three different measures (length, width, height), each individually representing a different plane. The epigynal and reproductive area were calculated from two measurements (length and width). We calculated SSD as mean female to male somatic character ratio, and SGD as mean female to male genital character ratio [10].

### Phylogenetic correlation analyses

To test pairwise correlations between measured characters, we used phylogenetically independent contrasts (PIC) [44] in the PDAP package of Mesquite. PIC requires a resolved phylogeny with specified branch lengths and assumes that evolutionary changes follow a Brownian motion model [45, 46]. We therefore used the most recent species-level phylogeny of nephilids and outgroups [40]. We used Nee’s branch length transformation in Mesquite that rendered all but three characters to conform to PDAP assumptions (Additional file 2: Table S1). The exceptions, two female somatic characters (body length and carapace width) and one male somatic character (carapace length), were not considered in correlational analyses. We checked for phylogenetic correlation between pairs of traits in PDAP using the option “Y contrasts vs. X contrasts (positivized)”. We deemed 2-tailed *p*-values to be significant at or below 0.05.

Additional file 3: Matrix S1 contains raw data, phylogenetic trees, and PDAP analyses.

### Phylogenetic generalized linear models

We examined correlations among traits in order to group correlated traits. We performed factor analyses [47] in each trait group (female somatic size, female external genital size, female internal genital size, male somatic size, male non-intromittent genital size, male intromittent genital size, SSD, SGD) using ‘fa’ function in R package ‘psych’ [48]. The results of factor analyses showed that, in most cases, the size measures from trait groups can be described by a single factor, except for female external genital size (Additional file 4: Appendix S1). We therefore used one factor to represent all the measurements in each trait group, except for female external genital size that was represented by factor one (copulatory opening width) and factor two that included the other three measurements (reproductive area, epigynal area, distance between copulatory openings). We then tested the correlation among these factors, and the analysis showed moderate to high correlations between them (Additional file 4: Appendix S1), which precluded our use of multiple linear regression due to collinearity. Therefore, we ran several phylogenetic generalized linear models (MCMCglmm) [49, 50] to examine the pairwise correlations among factors using the R package ‘MCMCglmm’ [51]. Using the original phylogeny (with non-transformed branch lengths) as a covariate, and the MCMCglmm settings: family=”gaussian” (for details and R code, see Additional file 5: Appendix S2), we ran 10 million generations, sampling every 200 generation, and discarding 25 % of generations as burnin.

## Results

In the 14 investigated nephilid species, the SSD values fell between 1.66 and 11.57, and SGD values were between 0.40 and 4.31. Out of 351 total pairwise comparisons, 99 comparisons showed significant correlation (Additional file 2: Table S1). After reducing the total number of comparisons to trait groups suggested by factor analyses, we explored broader correlation patterns through 36 pairwise comparisons between factors. Of these, ten were significantly correlated (Table 1; Additional file 5: Appendix S2).

**Table 1:** Results of MCMCglmm analyses testing correlation between trait groups. For details, see Additional file 5: Appendix S2

	Female somatic size	Female external genital size	Female copulatory opening width	Female internal genital size	Male somatic size	Male non-intromittent genital size	Male intromittent genital size	SSD	SGD
Female somatic size	-								
Female external genital size	0.689*	-							
Female copulatory opening width	ns	ns	-						
Female internal genital size	ns	ns	ns	-					
Male somatic size	ns	ns	ns	ns	-				
Male non-intromittent enital size	ns	0.600*	ns	ns	0.573**	-			
Male intromittent genital size	0.725*	0.809***	38.028*	ns	ns	0.594*	-		
SSD	0.871***	ns	ns	ns	ns	ns	ns	-	
SGD	ns	0.922***	ns	ns	ns	ns	0.655*	ns	-

Significance levels: *ns* non significant; *< 0.05; **< 0.01; ***< 0.001. Slope values are given for the significant results

### Pairwise correlations within sexes

Within each sex, all body size measures were significantly correlated to each other (Additional file 2: Table S1). Pairwise correlation analyses within a sex and between mixed characters (Additional file 2: Table S1), i.e. between a somatic and a genital character, revealed that female genital size measures (both external and internal measures) did not correlate with female somatic characters, albeit with one exception - reproductive duct length vs. female first tibia + patella length. Most of the female internal genital measures correlated with external genital measures (Additional file 2: Table S1). In contrast, MCMCglmm analyses detected female somatic size to be positively correlated to the factor that grouped most features of female external genital size (not copulatory opening width), but found no correlation between the latter factor and internal genital size (Table 1).

In males, non-intromittent genital size measures (embolic conductor length, bulb and pedipalp volume) significantly positively correlated with total male body length (and certain other measures of somatic size). However, male intromittent genital size measures (embolus width and volume) did not correlate with body size, but in part correlated with non-intromittent genital size (Additional file 2: Table S1). MCMCglmm analyses corroborate these patterns by showing that non-intromittent genital size significantly positively correlated with somatic size and intromittent genital size, but intromittent genital size and somatic size were independent (Table 1).

### Pairwise correlations between sexes

None of the somatic size traits were correlated between the sexes (Additional file 2: Table S1) corroborating the outcomes of prior studies of nephilid size evolution that only used total body length [13, 29]. Likewise, somatic size generally did not correlate with genital size of the opposite sex, with the exception of female copulatory duct width and male intromittent genital characters (Additional file 2: Table S1). While the male intromittent genital characters were correlated to female external and some internal genital features (spermathecal volume), the non-intromittent male genital characters were only correlated with one internal (spermathecal volume) and one external female genital trait (distance between copulatory openings) (Additional file 2: Table S1).

Similarly, MCMCglmm analyses revealed no correlations between male somatic size and female external and internal genital size factors, or with female somatic size (Table 1). However, male intromittent genital size was positively correlated with female somatic and external genital size (including copulatory opening width), but not internal genital size (Table 1). On the other hand, male non-intromittent genital size was only correlated with the female external genital size factor (Table 1).

### No correlation between SSD and SGD

Measures of SGD showed no correlation with any female or male body size trait (Additional file 2: Table S1). Likewise, SSD was not correlated to any male or female genital size measure. Finally, SSD and SGD variables were not correlated in any pairwise comparison using any metric (Additional file 2: Table S1). The MCMCglmm analyses agreed with pairwise correlation analyses (Table 1).

## Discussion

**Fig. 3:**
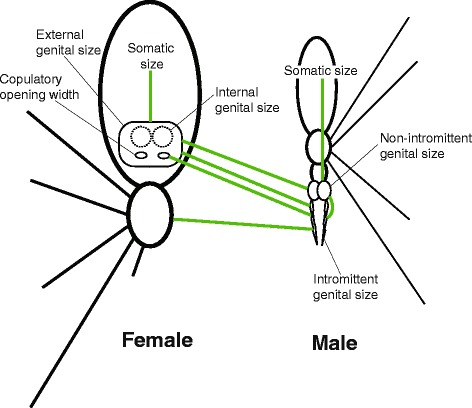
Main significant positive correlations between groups of traits as detected by the MCMCglmm analyses. Simplified scheme of male and female spiders in ventral view (not to scale)

**Fig. 4:**
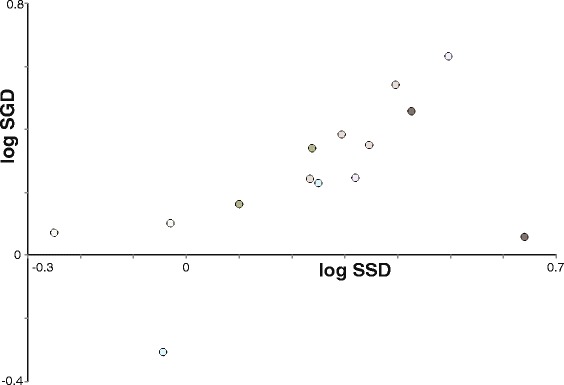
Detected scatter between SSD and SGD. No significant relationship between SSD and SGD, and the scattered SGD values combined provide no support for any of the theoretical models in Fig. 1. Data points are color coded after the phylogeny in Additional file 1: Figure S1

All analyses agree that internal female genital size is independent of female somatic size. Likewise, male intromittent genital size is independent of male somatic size, and correlates with female external, but not internal genital size (Fig. 3). On the other hand, male non-intromittent genital size correlates with male somatic size. Contrary to the previous study [10], we find no support for any of the theoretical models explaining the relationship between SSD and SGD (Fig. 1). Against prediction, we detected no phylogenetic correlation between SGD and SSD and scattered values for SGD (Fig. 4), a pattern suggesting that genital and somatic size dimorphisms evolve independently.

Detecting no phylogenetic correlation between female internal genital and body sizes suggests that these traits are under different selection pressures. In spiders, female body size is generally believed to be under strong positive fecundity selection [38], whereas female genitalia may respond to diverse aspects of sexual selection [35, 52, 53], or to natural selection that counters genital misfits [35]. On the other hand, we detected a positive correlation between male non-intromittent genital size and male body size, implying that the same selection pressures that act on male size also drive the size of non-intromittent part of the genitalia. The evolution of male body size in spiders is less understood than that of females, and is interpreted to respond to a combination of natural and sexual selection drivers, e.g. to female choice [31], male-male competition [54], sexual cannibalism [55], protandry [56, 57], differential mortality [58], or gravity [59–61]. The detected correlations (Fig. 3) imply that these mechanisms also shape the changes in the size of non-intromittent, but not also intromittent part of the male pedipalp.

Different components of male genitalia, such as the intromittent (embolus) and non-intromittent parts (other pedipalp components), play different roles during mating [62]. The embolus is the sclerite that penetrates into female internal genital ducts and the spermathecae, while other palpal sclerites help position and hook the palp as well as rotate it, and thus interact with female external genital components. From the functional perspective, it is thus logical that these components should respond to different selection pressures and therefore evolve independently. In water striders, for example, the evolution of non-intromittent genital characters is driven by premating selection whereas the evolution of intromittent genital characters is affected by post-mating sexual selection [63, 64]. Here, we provide evidence that the size of non-intromittent male genital components coevolves with male body size. Such pattern is rarely detected in invertebrates [6, 65], and is new in spiders.

We found evidence for a strongly correlated size evolution between male intromittent genital parts and female external genitalia, including copulatory opening width (Fig. 3). This pattern suggests size coevolution of those components of genitalia that interact during mating, i.e. intromittent male genital parts (the embolus) with female copulatory openings and in part the female internal genitals. While the size correlation between the embolus and the parts of female internal genitals only holds for spermathecal volume (but not the size of the insemination ducts; Additional file 2: Table S1), the correlations between the embolus size and every single component of external female genital aparatus (including copulatory opening width, the distance between them, as well as epigynal and reproductive area) suggest that the size evolution of these components acts directly against genital size mismatches that would otherwise arise as a consequence of SSD.

Ramos et al. [10] found support for the positive genital divergence model looking broadly into orb web spiders. However, our analyses fail to support that or any other proposed evolutionary model (Fig. 1). The lack of any correlation between SSD and SGD falsifies the positive and the negative genital divergence model, and scattered SGD values furthermore reject the constant model (Figs. 1 and 4). These results support our initial prediction that genital and somatic size dimorphism are phylogenetically decoupled, i.e. traits composing genital versus somatic size dimorphism evolve independently. Since most spiders possess heavily sclerotized genitalia that do not adjust shape and size during copulation, any size misfits would be selected against. There is profound theoretical basis for this assumption, and both natural and sexual selection mechanisms predict selection for size and anatomical match between male and female genitalia [6, 21, 35, 66]. If somatic sizes evolve mismatches (SSD), then the smaller sex (males) may be expected to compensate in genital size. Furthermore, the effectiveness of male genital plugs, as known in nephilid spiders, would be compromised in the case of considerable genital size mismatch between the sexes [33, 67] because male genital plugs could be bypassed by emboli of rivals. The picture is complicated because both intromittent and non-intromittent palpal parts may be seen as contributors to SGD, but it seems evident that the intromittent parts compensate in size evolution in order to avoid genital size mismatches.

Although we discuss the independent evolution of those traits that do not show phylogenetic correlation, due to the number of species investigated (14) it may be that our study was only able to detect the strongest correlations between traits/factors.

## Conclusions

We show that the internal dimensions of female genitalia evolve independently of female body size in nephilid spiders. Likewise, we show that male intromittent genital size evolves independently of the male body size. We hypothesize that through functional interaction, the size of the male intromittent organ, the embolus, and the sizes of female internal and external genital components, respond to selection against genital size mismatches that would otherwise be inevitable considering SSD levels. We conclude that genital size and somatic size are independent components of sexual size dimorphism, a result that rejects prior theoretical models of size evolution.

## Additional files

Additional file 1: Figure S1. Simplified phylogeny of nephilid spiders highlighting the investigated species representing genus-level clades. Phylogeny depicts the consensus Bayesian tree [40]. (PDF 10 kb) Additional file 2: Table S1. Phylogenetic correlation results from PDAP for all pairs of parameters. Abbreviations: FTPL: Female Tibia-Patella Length. FCL: Female Carapace Length.sq_RA: square-root Reproductive Area. sq_EA: square-root Epigynal Area. DBO: distance between copulatory openings. open_W: copulatory opening Width. duct_L: reproductive duct Length. cube_SV: cube-root Spermatheca Volume. MBL: Male Body Length. MTPL: Male Tibia-Patella Length. MCW: Male Carapace Width. MCL: Male Carapace Length. ECL: Embolic Conductor Length. cube_BV: cube-root pedipal Bulb Volume. cube_PV: cube-root pedipalp Volume. EW: Embolus Width. cube_EV: cube-root embolus Volume. SSD_BL: FBL/MBL. SSD_TPL: FTPL/MTPL. SSD_CW: FCW/MCW. SSD_CL: FCL/MCL. SGD_SB: Spermatheca volume/Pedipalp Bulb volume. SGD_SP: Spermatheca volume/Pedipalp volume. SGD_EB: Epigynal area/Pedipalp Bulb volume. SGD_EP: Epigynal area/Pedipalp volume. SGD_RB: Reproductive Area/Pedipalp Bulb volume. SGD_RP: Reproductive Area/Pedipalp volume. (XLSX 32 kb) Additional file 3: Matrix S1. A nexus file containing raw data, phylogenetic trees, and PDAP analyses. (TXT 81 kb) Additional file 4: Appendix S1. The R code and the results of factor analysis. (DOCX 119 kb) Additional file 5: Appendix S2. The R code and the results of MCMCglmm analyses. (DOCX 36 kb)
