# Supplementary figures and images for: Coevolution of female and male genital components to avoid genital size mismatches in sexually dimorphic spiders
Source: BMC Evol Biol. 2016 Aug 17;16:161. doi: 10.1186/s12862-016-0734-9 (PMC4989301; doi:10.1186/s12862-016-0734-9)

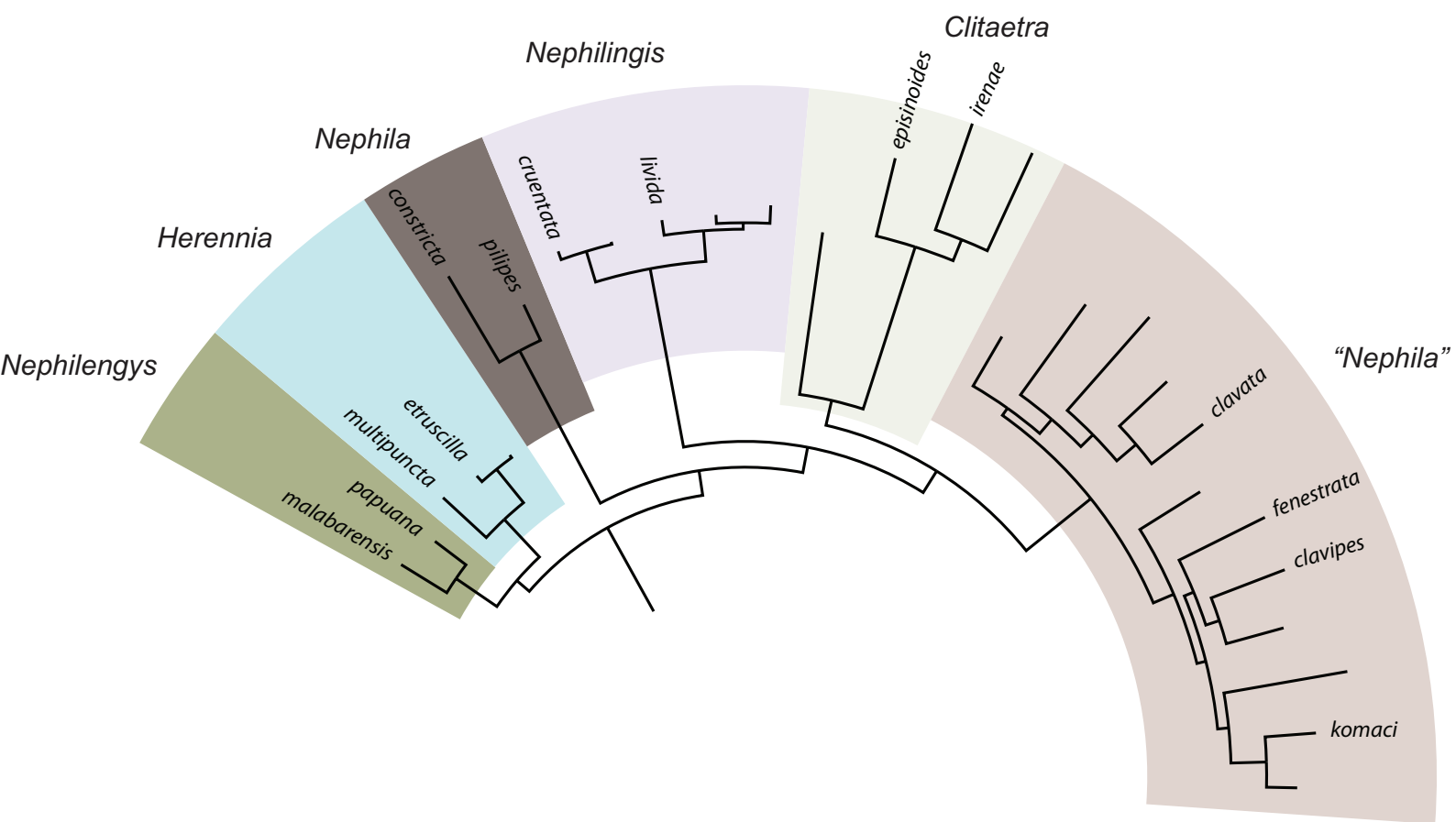

Supplement: Additional file 1: Figure S1. — Simplified phylogeny of nephilid spiders highlighting the investigated species representing genus-level clades. Phylogeny depicts the consensus Bayesian tree [40]. (PDF 10 kb) [file 12862_2016_734_MOESM1_ESM.pdf]
