# Supplementary material for: Coevolution of female and male genital components to avoid genital size mismatches in sexually dimorphic spiders
Source: BMC Evol Biol. 2016 Aug 17;16:161. doi: 10.1186/s12862-016-0734-9 (PMC4989301; doi:10.1186/s12862-016-0734-9)

## read data

d <- read.table("FA.txt",header=T)

d$phylo <- as.factor(d$phylo)

contrasts(d$phylo) <- contr.sum

## scatter plot

panel.cor <- function(x, y, digits = 2, prefix = "", cex.cor, ...){

usr <- par("usr"); on.exit(par(usr))

par(usr = c(0, 1, 0, 1))

ra <- abs(cor(x, y, method="pearson"))

r <- cor(x, y, method="pearson")

txt <- format(c(r, 0.123456789), digits = digits)[1]

txt <- paste0(prefix, txt)

if(missing(cex.cor)) cex.cor <- 1.5/strwidth(txt)

text(0.5, 0.5, txt, cex = ra*1.2+0.5)

}

panel.smooth <- function (x, y, col = par("col"), bg = NA, pch = par("pch"),

cex = 1, col.smooth = "red", span = 4/5, iter = 10, ...)

{

points(x, y, pch = pch, col = col, bg = bg, cex = cex)

ok <- is.finite(x) & is.finite(y)

if (any(ok))

lines(stats::lowess(x[ok], y[ok], f = span, iter = iter),

col = col.smooth, ...)

}

pairs(

d[, -1],

lower.panel=panel.smooth,

upper.panel=panel.cor, pch=20, col="#000000", cex=0.2, gap=0.2

)

## Factor analysis

d.pred.group <- data.frame(phylo = d$phylo)

## Female somatic size

factorNumber <- 1

dat_FB <- d[c(2:5)]

fa.FB <- fa(dat_FB, factorNumber, rotate="oblimin") %>% fa.sort

fa.FB; KMO(scores(fa.FB)); cortest.bartlett(scores(fa.FB))

dev.new(); fa.diagram(fa.FB, simple=F, side=4, cex=9/12)

dev.new(); plot(fa.FB, xlim=c(0,5), ylim=c(0,5))

dev.new(); fa.parallel(dat_FB, n.iter=50, error.bars=T, fa="fa")

d.pred.group$FB <- fa.FB %>% scores %>% .[,1]

## Female external genital size

factorNumber <- 1

dat_FE <- d[c(6:8)]

fa.FE <- fa(dat_FE, factorNumber, rotate="oblimin") %>% fa.sort

fa.FE; KMO(scores(fa.FE)); cortest.bartlett(scores(fa.FE))

dev.new(); fa.diagram(fa.FE, simple=F, side=4, cex=9/12)

dev.new(); plot(fa.FE, xlim=c(0,5), ylim=c(0,5))

dev.new(); fa.parallel(dat_FE, n.iter=50, error.bars=T, fa="fa")

d.pred.group$FE <- fa.FE %>% scores %>% .[,1]

d.pred.group$open_W <- d$open_W

## Female internal genital size

factorNumber <- 1

dat_FI <- d[c(10:12)]

fa.FI <- fa(dat_FI, factorNumber, rotate="oblimin") %>% fa.sort

fa.FI; KMO(scores(fa.FI)); cortest.bartlett(scores(fa.FI))

dev.new(); fa.diagram(fa.FI, simple=F, side=4, cex=9/12)

dev.new(); plot(fa.FI, xlim=c(0,5), ylim=c(0,5))

dev.new(); fa.parallel(dat_FI, n.iter=50, error.bars=T, fa="fa")

d.pred.group$FI <- fa.FI %>% scores %>% .[,1]

## Male somatic size

factorNumber <- 1

dat_MB <- d[c(13:16)]

fa.MB <- fa(dat_MB, factorNumber, rotate="oblimin") %>% fa.sort

fa.MB; KMO(scores(fa.MB)); cortest.bartlett(scores(fa.MB))

dev.new(); fa.diagram(fa.MB, simple=F, side=4, cex=9/12)

dev.new(); plot(fa.MB, xlim=c(0,5), ylim=c(0,5))

dev.new(); fa.parallel(dat_MB, n.iter=50, error.bars=T, fa="fa")

d.pred.group$MB <- fa.MB %>% scores %>% .[,1]

## Male non-intromittent genital size

factorNumber <- 1

dat_MNI <- d[c(17:19)]

fa.MNI <- fa(dat_MNI, factorNumber, rotate="oblimin") %>% fa.sort

fa.MNI; KMO(scores(fa.MNI)); cortest.bartlett(scores(fa.MNI))

dev.new(); fa.diagram(fa.MNI, simple=F, side=4, cex=9/12)

dev.new(); plot(fa.MNI, xlim=c(0,5), ylim=c(0,5))

dev.new(); fa.parallel(dat_MNI, n.iter=50, error.bars=T, fa="fa")

d.pred.group$MNI <- fa.MNI %>% scores %>% .[,1]

## Male intromittent genital size

factorNumber <- 1

dat_MI <- d[c(20:21)]

fa.MI <- fa(dat_MI, factorNumber, rotate="oblimin") %>% fa.sort

fa.MI; KMO(scores(fa.MI)); cortest.bartlett(scores(fa.MI))

dev.new(); fa.diagram(fa.MI, simple=F, side=4, cex=9/12)

dev.new(); plot(fa.MI, xlim=c(0,5), ylim=c(0,5))

dev.new(); fa.parallel(dat_MI, n.iter=50, error.bars=T, fa="fa")

d.pred.group$MI <- fa.MI %>% scores %>% .[,1]

## SSD

factorNumber <- 1

dat_SSD <- d[c(22:25)]

fa.SSD <- fa(dat_SSD, factorNumber, rotate="oblimin") %>% fa.sort

fa.SSD; KMO(scores(fa.SSD)); cortest.bartlett(scores(fa.SSD))

dev.new(); fa.diagram(fa.SSD, simple=F, side=4, cex=9/12)

dev.new(); plot(fa.SSD, xlim=c(0,5), ylim=c(0,5))

dev.new(); fa.parallel(dat_SSD, n.iter=50, error.bars=T, fa="fa")

d.pred.group$SSD <- fa.SSD %>% scores %>% .[,1]

## SGD

factorNumber <- 1

dat_SGD <- d[c(26:31)]

fa.SGD <- fa(dat_SGD, factorNumber, rotate="oblimin") %>% fa.sort

fa.SGD; KMO(scores(fa.SGD)); cortest.bartlett(scores(fa.SGD))

dev.new(); fa.diagram(fa.SGD, simple=F, side=4, cex=9/12)

dev.new(); plot(fa.SGD, xlim=c(0,5), ylim=c(0,5))

dev.new(); fa.parallel(dat_SGD, n.iter=50, error.bars=T, fa="fa")

d.pred.group$SGD <- fa.SGD %>% scores %>% .[,1]

write.csv(d.pred.group, file="Nep_Gential_FA.csv", row.names = F)

pairs(data,

lower.panel=panel.smooth,

upper.panel=panel.cor, pch=20, col="#000000", cex=0.2, gap=0.2)

# FA_FB

Factor Analysis using method = minres

Call: fa(r = dat_FB, nfactors = factorNumber, rotate = "oblimin")

Standardized loadings (pattern matrix) based upon correlation matrix

MR1 h2 u2 com

FBL 0.98 0.97 0.032 1

FCW 0.98 0.97 0.033 1

FCL 0.98 0.97 0.035 1

FTPL 0.94 0.89 0.110 1

MR1

SS loadings 3.79

Proportion Var 0.95

Mean item complexity = 1

Test of the hypothesis that 1 factor is sufficient.

The degrees of freedom for the null model are 6 and the objective function was 8.97 with Chi Square of 97.16

The degrees of freedom for the model are 2 and the objective function was 1.37

The root mean square of the residuals (RMSR) is 0.03

The df corrected root mean square of the residuals is 0.05

The harmonic number of observations is 14 with the empirical chi square 0.12 with prob < 0.94

The total number of observations was 14 with MLE Chi Square = 13.89 with prob < 0.00097

Tucker Lewis Index of factoring reliability = 0.581

RMSEA index = 0.778 and the 90 % confidence intervals are 0.358 0.994

BIC = 8.61

Fit based upon off diagonal values = 1

Measures of factor score adequacy

MR1

Correlation of scores with factors 1.00

Multiple R square of scores with factors 0.99

Minimum correlation of possible factor scores 0.99

Kaiser-Meyer-Olkin factor adequacy

Call: KMO(r = scores(fa.FB))

Overall MSA = NaN

MSA for each item =

MR1

NaN

R was not square, finding R from data

$chisq

[1] 0

$p.value

[1] 1

$df

[1] 0


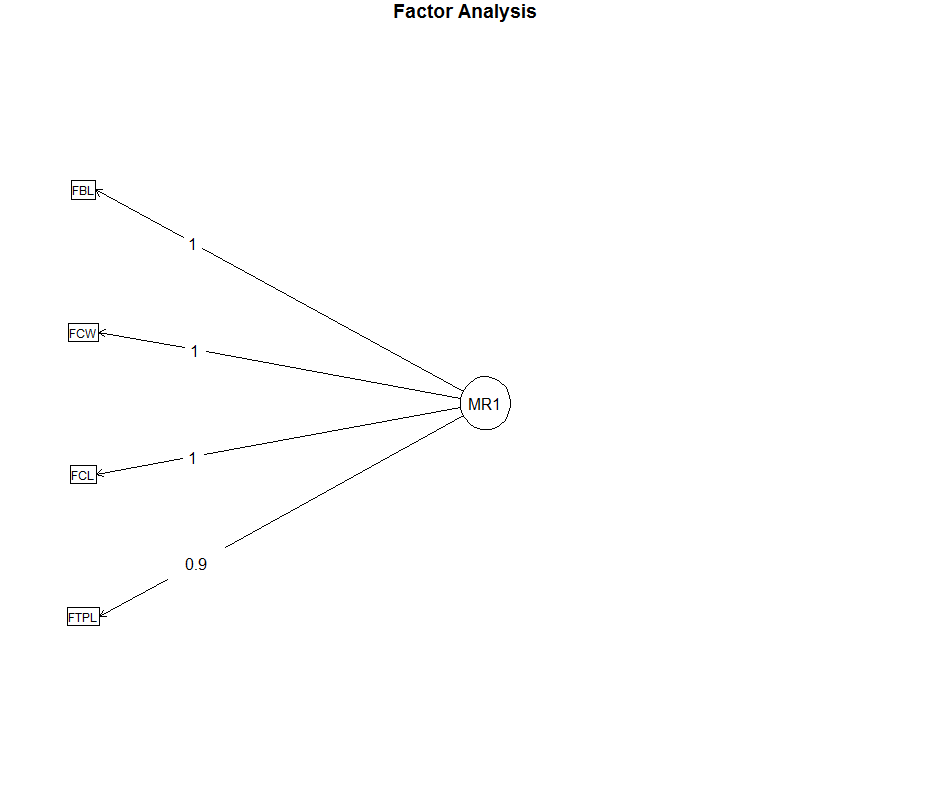


# FA_FE

Factor Analysis using method = minres

Call: fa(r = dat_FE, nfactors = factorNumber, rotate = "oblimin")

Standardized loadings (pattern matrix) based upon correlation matrix

MR1 h2 u2 com

sq_EA 0.97 0.94 0.060 1

sq_RA 0.97 0.94 0.064 1

DBO 0.89 0.79 0.210 1

MR1

SS loadings 2.67

Proportion Var 0.89

Mean item complexity = 1

Test of the hypothesis that 1 factor is sufficient.

The degrees of freedom for the null model are 3 and the objective function was 3.56 with Chi Square of 39.79

The degrees of freedom for the model are 0 and the objective function was 0

The root mean square of the residuals (RMSR) is 0

The df corrected root mean square of the residuals is NA

The harmonic number of observations is 14 with the empirical chi square 0 with prob < NA

The total number of observations was 14 with MLE Chi Square = 0 with prob < NA

Tucker Lewis Index of factoring reliability = -Inf

Fit based upon off diagonal values = 1

Measures of factor score adequacy

MR1

Correlation of scores with factors 0.99

Multiple R square of scores with factors 0.97

Minimum correlation of possible factor scores 0.94

Kaiser-Meyer-Olkin factor adequacy

Call: KMO(r = scores(fa.FE))

Overall MSA = NaN

MSA for each item =

MR1

NaN

R was not square, finding R from data

$chisq

[1] 0

$p.value

[1] 1

$df

[1] 0


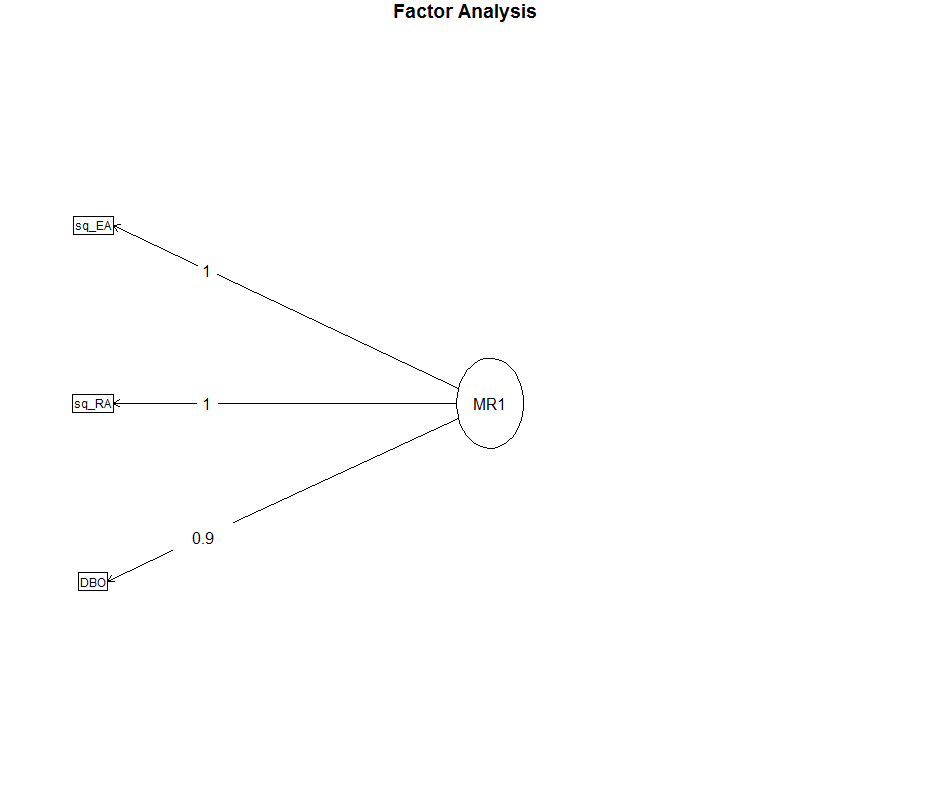


# FA_FI

Factor Analysis using method = minres

Call: fa(r = dat_FI, nfactors = factorNumber, rotate = "oblimin")

Standardized loadings (pattern matrix) based upon correlation matrix

MR1 h2 u2 com

duct_L 1.00 1.00 0.005 1

cube_SV 0.61 0.37 0.634 1

duct_W 0.52 0.27 0.725 1

MR1

SS loadings 1.64

Proportion Var 0.55

Mean item complexity = 1

Test of the hypothesis that 1 factor is sufficient.

The degrees of freedom for the null model are 3 and the objective function was 0.78 with Chi Square of 8.74

The degrees of freedom for the model are 0 and the objective function was 0.01

The root mean square of the residuals (RMSR) is 0.04

The df corrected root mean square of the residuals is NA

The harmonic number of observations is 14 with the empirical chi square 0.12 with prob < NA

The total number of observations was 14 with MLE Chi Square = 0.1 with prob < NA

Tucker Lewis Index of factoring reliability = -Inf

Fit based upon off diagonal values = 0.99

Measures of factor score adequacy

MR1

Correlation of scores with factors 1.00

Multiple R square of scores with factors 1.00

Minimum correlation of possible factor scores 0.99

Kaiser-Meyer-Olkin factor adequacy

Call: KMO(r = scores(fa.FI))

Overall MSA = NaN

MSA for each item =

MR1

NaN

R was not square, finding R from data

$chisq

[1] 0

$p.value

[1] 1

$df

[1] 0


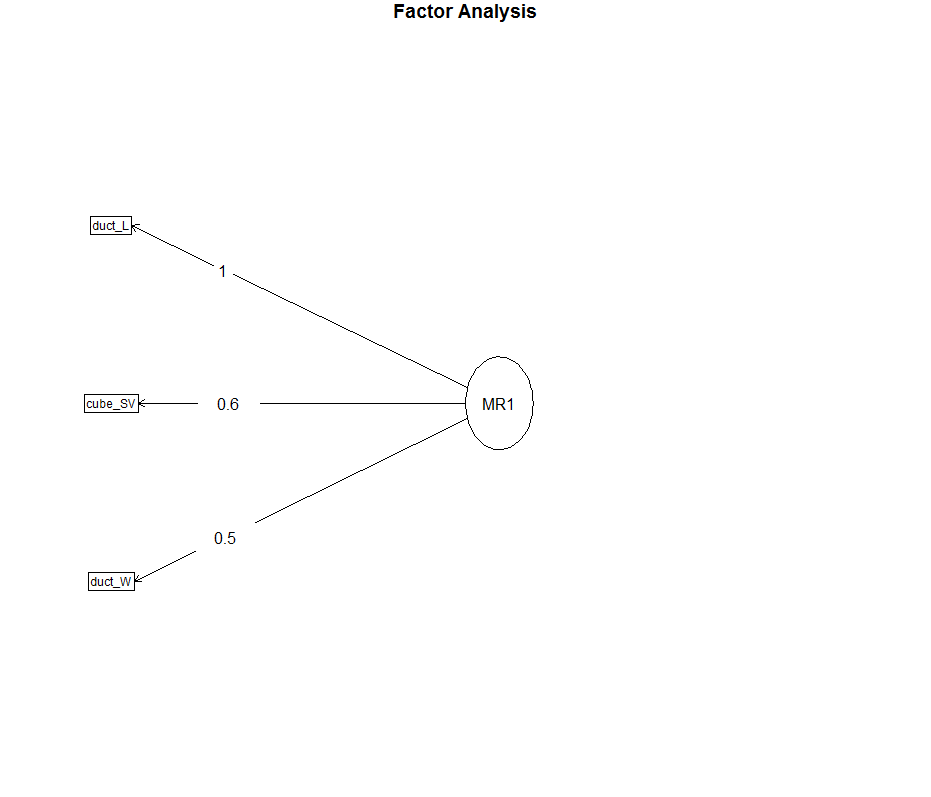


# FA_MB

Factor Analysis using method = minres

Call: fa(r = dat_MB, nfactors = factorNumber, rotate = "oblimin")

Standardized loadings (pattern matrix) based upon correlation matrix

MR1 h2 u2 com

MCL 0.96 0.93 0.073 1

MCW 0.94 0.89 0.113 1

MBL 0.85 0.72 0.278 1

MTPL 0.75 0.56 0.439 1

MR1

SS loadings 3.10

Proportion Var 0.77

Mean item complexity = 1

Test of the hypothesis that 1 factor is sufficient.

The degrees of freedom for the null model are 6 and the objective function was 5.15 with Chi Square of 55.8

The degrees of freedom for the model are 2 and the objective function was 1.47

The root mean square of the residuals (RMSR) is 0.12

The df corrected root mean square of the residuals is 0.21

The harmonic number of observations is 14 with the empirical chi square 2.48 with prob < 0.29

The total number of observations was 14 with MLE Chi Square = 14.93 with prob < 0.00057

Tucker Lewis Index of factoring reliability = 0.164

RMSEA index = 0.811 and the 90 % confidence intervals are 0.386 1.02

BIC = 9.65

Fit based upon off diagonal values = 0.98

Measures of factor score adequacy

MR1

Correlation of scores with factors 0.98

Multiple R square of scores with factors 0.96

Minimum correlation of possible factor scores 0.93

Kaiser-Meyer-Olkin factor adequacy

Call: KMO(r = scores(fa.MB))

Overall MSA = NaN

MSA for each item =

MR1

NaN

R was not square, finding R from data

$chisq

[1] 0

$p.value

[1] 1

$df

[1] 0


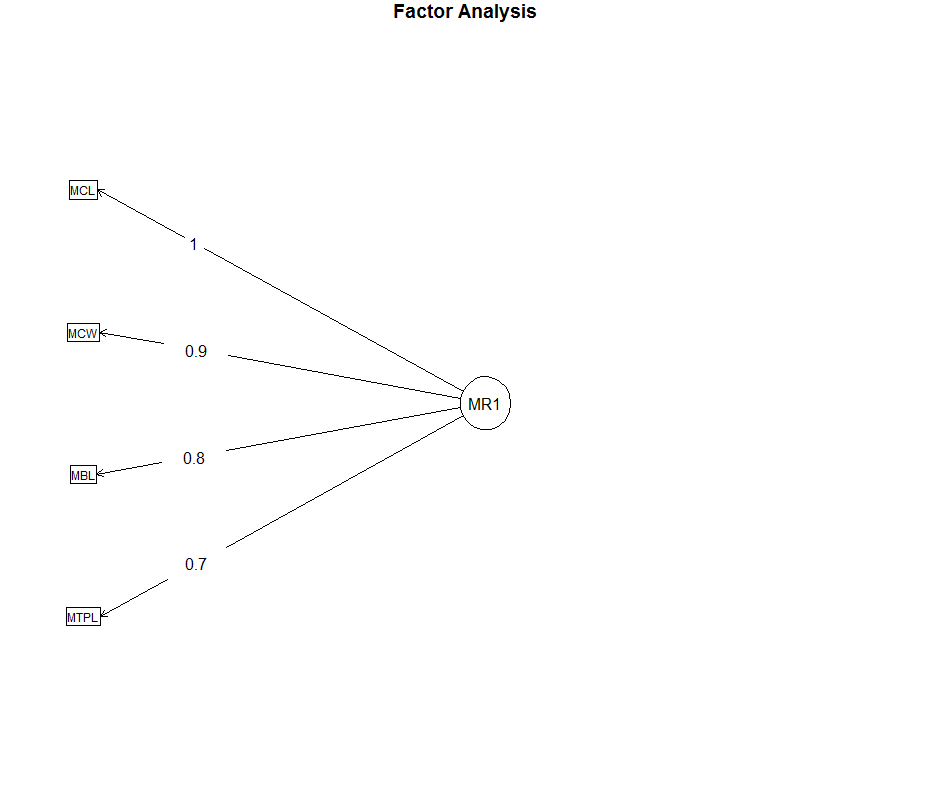


# FA_MNI

Factor Analysis using method = minres

Call: fa(r = dat_MNI, nfactors = factorNumber, rotate = "oblimin")

Standardized loadings (pattern matrix) based upon correlation matrix

MR1 h2 u2 com

cube_PV 1.00 1.00 0.0025 1

cube_BV 1.00 1.00 0.0026 1

ECL 0.69 0.48 0.5244 1

MR1

SS loadings 2.47

Proportion Var 0.82

Mean item complexity = 1

Test of the hypothesis that 1 factor is sufficient.

The degrees of freedom for the null model are 3 and the objective function was 10.24 with Chi Square of 114.36

The degrees of freedom for the model are 0 and the objective function was 3.36

The root mean square of the residuals (RMSR) is 0

The df corrected root mean square of the residuals is NA

The harmonic number of observations is 14 with the empirical chi square 0 with prob < NA

The total number of observations was 14 with MLE Chi Square = 35.29 with prob < NA

Tucker Lewis Index of factoring reliability = -Inf

Fit based upon off diagonal values = 1

Measures of factor score adequacy

MR1

Correlation of scores with factors 1

Multiple R square of scores with factors 1

Minimum correlation of possible factor scores 1

Kaiser-Meyer-Olkin factor adequacy

Call: KMO(r = scores(fa.MNI))

Overall MSA = NaN

MSA for each item =

MR1

NaN

R was not square, finding R from data

$chisq

[1] 0

$p.value

[1] 1

$df

[1] 0


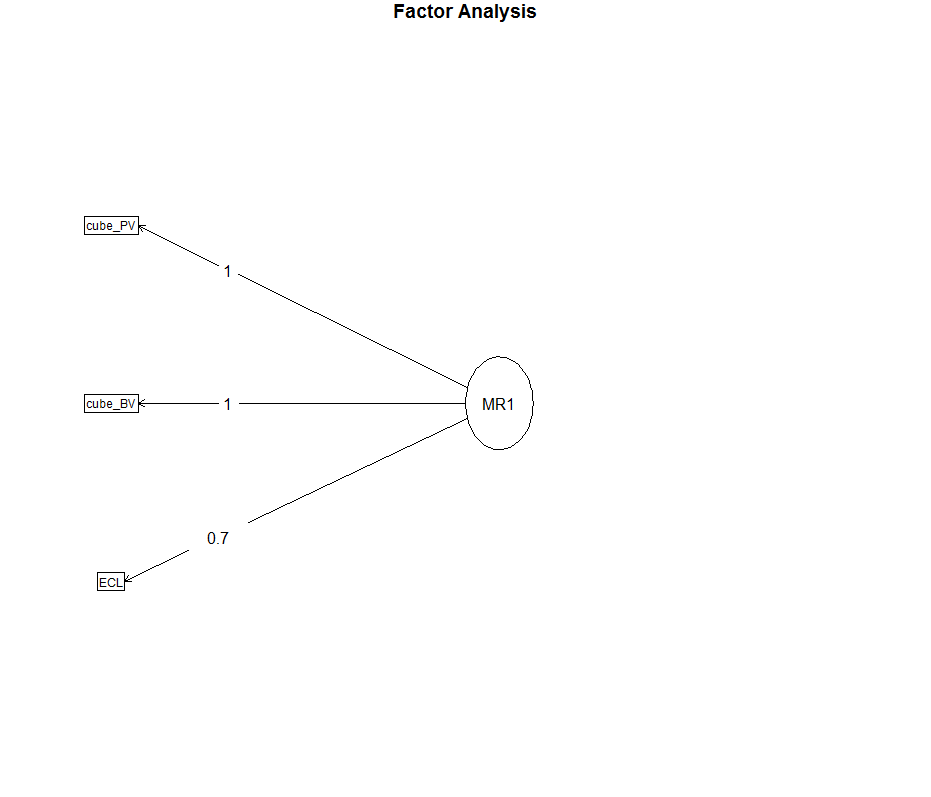


# FA_MI

Factor Analysis using method = minres

Call: fa(r = dat_MI, nfactors = factorNumber, rotate = "oblimin")

Standardized loadings (pattern matrix) based upon correlation matrix

MR1 h2 u2 com

EW 0.94 0.88 0.12 1

cube_EV 0.94 0.88 0.12 1

MR1

SS loadings 1.76

Proportion Var 0.88

Mean item complexity = 1

Test of the hypothesis that 1 factor is sufficient.

The degrees of freedom for the null model are 1 and the objective function was 1.51 with Chi Square of 17.34

The degrees of freedom for the model are -1 and the objective function was 0

The root mean square of the residuals (RMSR) is 0

The df corrected root mean square of the residuals is NA

The harmonic number of observations is 14 with the empirical chi square 0 with prob < NA

The total number of observations was 14 with MLE Chi Square = 0 with prob < NA

Tucker Lewis Index of factoring reliability = 1.065

Fit based upon off diagonal values = 1

Measures of factor score adequacy

MR1

Correlation of scores with factors 0.97

Multiple R square of scores with factors 0.94

Minimum correlation of possible factor scores 0.88

Kaiser-Meyer-Olkin factor adequacy

Call: KMO(r = scores(fa.MI))

Overall MSA = NaN

MSA for each item =

MR1

NaN

R was not square, finding R from data

$chisq

[1] 0

$p.value

[1] 1

$df

[1] 0


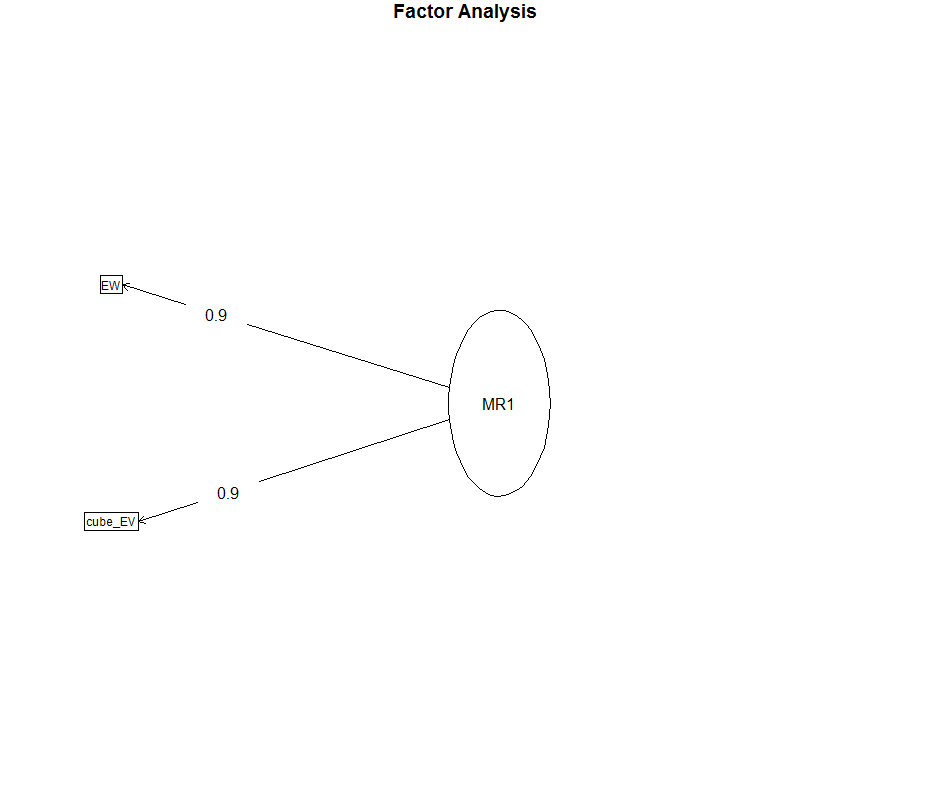


# FA_SSD

Factor Analysis using method = minres

Call: fa(r = dat_SSD, nfactors = factorNumber, rotate = "oblimin")

Standardized loadings (pattern matrix) based upon correlation matrix

MR1 h2 u2 com

SSD_CL 0.99 0.99 0.012 1

SSD_BL 0.99 0.98 0.018 1

SSD_CW 0.99 0.98 0.021 1

SSD_TPL 0.99 0.98 0.023 1

MR1

SS loadings 3.92

Proportion Var 0.98

Mean item complexity = 1

Test of the hypothesis that 1 factor is sufficient.

The degrees of freedom for the null model are 6 and the objective function was 10.95 with Chi Square of 118.59

The degrees of freedom for the model are 2 and the objective function was 0.26

The root mean square of the residuals (RMSR) is 0

The df corrected root mean square of the residuals is 0.01

The harmonic number of observations is 14 with the empirical chi square 0 with prob < 1

The total number of observations was 14 with MLE Chi Square = 2.65 with prob < 0.27

Tucker Lewis Index of factoring reliability = 0.981

RMSEA index = 0.231 and the 90 % confidence intervals are NA 0.575

BIC = -2.63

Fit based upon off diagonal values = 1

Measures of factor score adequacy

MR1

Correlation of scores with factors 1.00

Multiple R square of scores with factors 1.00

Minimum correlation of possible factor scores 0.99

Kaiser-Meyer-Olkin factor adequacy

Call: KMO(r = scores(fa.SSD))

Overall MSA = NaN

MSA for each item =

MR1

NaN

R was not square, finding R from data

$chisq

[1] 0

$p.value

[1] 1

$df

[1] 0


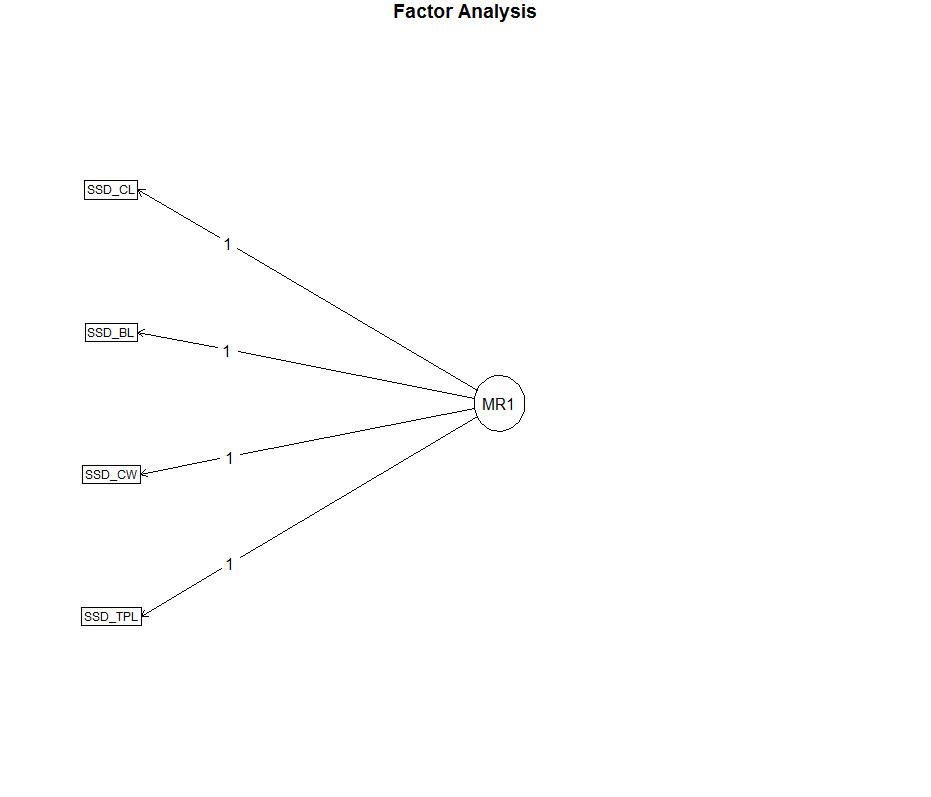


# FA_SGD

Factor Analysis using method = minres

Call: fa(r = dat_SGD, nfactors = factorNumber, rotate = "oblimin")

Standardized loadings (pattern matrix) based upon correlation matrix

MR1 h2 u2 com

SGD_RP 0.96 0.92 0.084 1

SGD_RB 0.96 0.92 0.084 1

SGD_EB 0.94 0.89 0.110 1

SGD_EP 0.94 0.89 0.110 1

SGD_SB 0.84 0.70 0.302 1

SGD_SP 0.83 0.70 0.303 1

MR1

SS loadings 5.01

Proportion Var 0.83

Mean item complexity = 1

Test of the hypothesis that 1 factor is sufficient.

The degrees of freedom for the null model are 15 and the objective function was 39.29 with Chi Square of 399.5

The degrees of freedom for the model are 9 and the objective function was 31.25

The root mean square of the residuals (RMSR) is 0.13

The df corrected root mean square of the residuals is 0.17

The harmonic number of observations is 14 with the empirical chi square 7.09 with prob < 0.63

The total number of observations was 14 with MLE Chi Square = 296.92 with prob < 1.2e-58

Tucker Lewis Index of factoring reliability = -0.339

RMSEA index = 1.843 and the 90 % confidence intervals are NA NA

BIC = 273.16

Fit based upon off diagonal values = 0.97

Measures of factor score adequacy

MR1

Correlation of scores with factors 1

Multiple R square of scores with factors 1

Minimum correlation of possible factor scores 1

Kaiser-Meyer-Olkin factor adequacy

Call: KMO(r = scores(fa.SGD))

Overall MSA = NaN

MSA for each item =

MR1

NaN

R was not square, finding R from data

$chisq

[1] 0

$p.value

[1] 1

$df

[1] 0


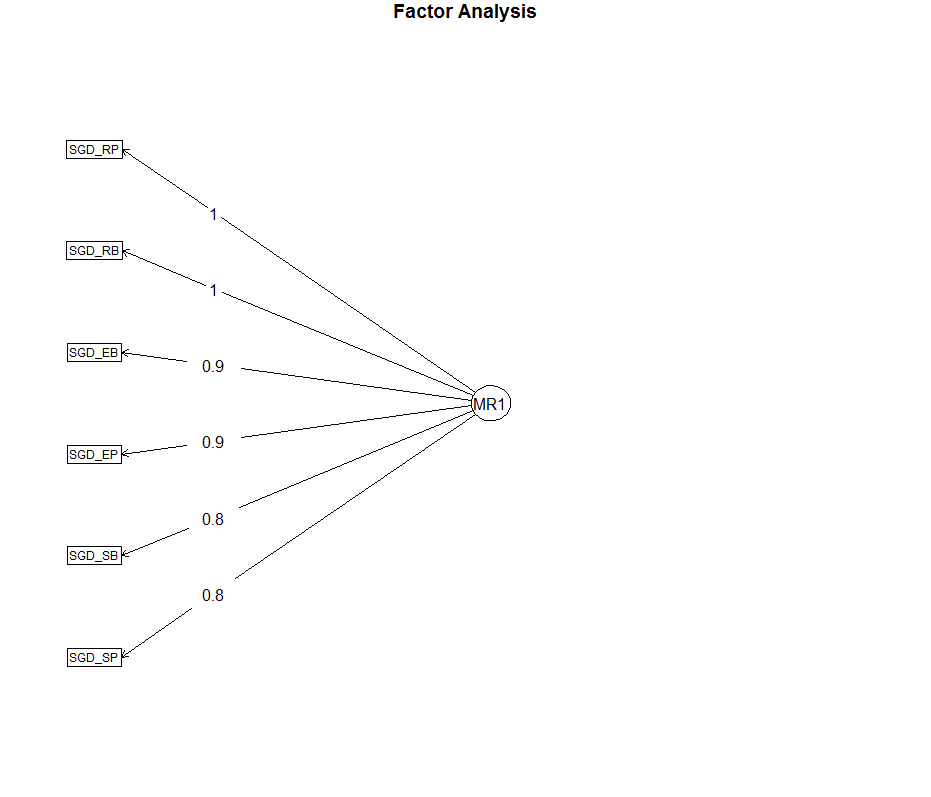


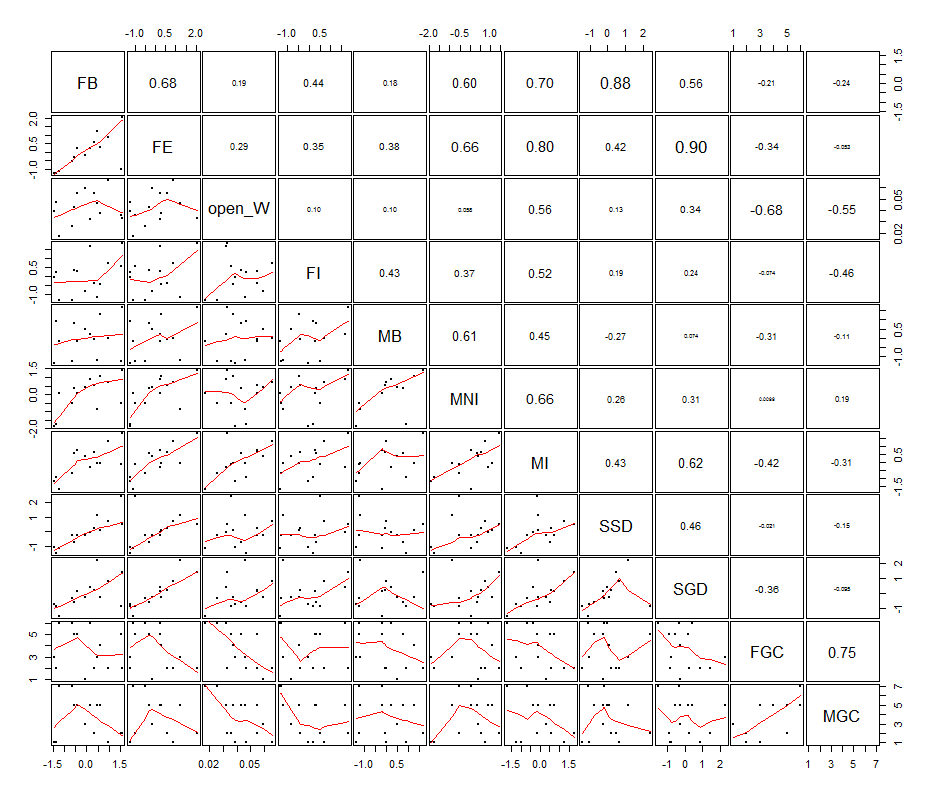

Supplement: Additional file 4: Appendix S1. — The R code and the results of factor analysis. (DOCX 119 kb) [file 12862_2016_734_MOESM4_ESM.docx]
