# Supplementary material for: Coevolution of female and male genital components to avoid genital size mismatches in sexually dimorphic spiders
Source: BMC Evol Biol. 2016 Aug 17;16:161. doi: 10.1186/s12862-016-0734-9 (PMC4989301; doi:10.1186/s12862-016-0734-9)
Supplement: Additional file 5: Appendix S2. — The R code and the results of MCMCglmm analyses. (DOCX 36 kb) [file 12862_2016_734_MOESM5_ESM.docx]

# MCMCglmm

phylo = read.nexus("Nee.nex")

plot(phylo)

data = read.table("Nep_Gential_FA.txt",header=T)

head(data)

# MCMCglmm_FB_FE

inv.phylo = inverseA(phylo,nodes="TIPS",scale=TRUE)

prior = list(G=list(G1=list(V=1,nu=0.02)),R=list(V=1,nu=0.02))

model_FB_FE<-MCMCglmm(FE~FB,random=~phylo,

family="gaussian",ginverse=list(phylo=inv.phylo$Ainv),prior=prior,data=data,nitt=10000000,burnin=2500000,thin=200)

summary(model_FB_FE)

mean(model_FB_FE$VCV[,2])

plot(model_FB_FE)

# MCMCglmm_FB_open_W

inv.phylo = inverseA(phylo,nodes="TIPS",scale=TRUE)

prior = list(G=list(G1=list(V=1,nu=0.02)),R=list(V=1,nu=0.02))

model_FB_open_W<-MCMCglmm(open_W~FB,random=~phylo,

family="gaussian",ginverse=list(phylo=inv.phylo$Ainv),prior=prior,data=data,nitt=10000000,burnin=2500000,thin=200)

summary(model_FB_open_W)

mean(model_FB_open_W$VCV[,2])

plot(model_FB_open_W)

# MCMCglmm_FB_FI

inv.phylo = inverseA(phylo,nodes="TIPS",scale=TRUE)

prior = list(G=list(G1=list(V=1,nu=0.02)),R=list(V=1,nu=0.02))

model_FB_FI<-MCMCglmm(FI~FB,random=~phylo,

family="gaussian",ginverse=list(phylo=inv.phylo$Ainv),prior=prior,data=data,nitt=10000000,burnin=2500000,thin=200)

summary(model_FB_FI)

mean(model_FB_FI$VCV[,2])

plot(model_FB_FI)

# MCMCglmm_FB_MB

inv.phylo = inverseA(phylo,nodes="TIPS",scale=TRUE)

prior = list(G=list(G1=list(V=1,nu=0.02)),R=list(V=1,nu=0.02))

model_FB_MB<-MCMCglmm(MB~FB,random=~phylo,

family="gaussian",ginverse=list(phylo=inv.phylo$Ainv),prior=prior,data=data,nitt=10000000,burnin=2500000,thin=200)

summary(model_FB_MB)

mean(model_FB_MB$VCV[,2])

plot(model_FB_MB)

# MCMCglmm_FB_MNI

inv.phylo = inverseA(phylo,nodes="TIPS",scale=TRUE)

prior = list(G=list(G1=list(V=1,nu=0.02)),R=list(V=1,nu=0.02))

model_FB_MNI<-MCMCglmm(MNI~FB,random=~phylo,

family="gaussian",ginverse=list(phylo=inv.phylo$Ainv),prior=prior,data=data,nitt=10000000,burnin=2500000,thin=200)

summary(model_FB_MNI)

mean(model_FB_MNI$VCV[,2])

plot(model_FB_MNI)

# MCMCglmm_FB_MI

inv.phylo = inverseA(phylo,nodes="TIPS",scale=TRUE)

prior = list(G=list(G1=list(V=1,nu=0.02)),R=list(V=1,nu=0.02))

model_FB_MI<-MCMCglmm(MI~FB,random=~phylo,

family="gaussian",ginverse=list(phylo=inv.phylo$Ainv),prior=prior,data=data,nitt=10000000,burnin=2500000,thin=200)

summary(model_FB_MI)

mean(model_FB_MI$VCV[,2])

plot(model_FB_MI)

# MCMCglmm_FB_SSD

inv.phylo = inverseA(phylo,nodes="TIPS",scale=TRUE)

prior = list(G=list(G1=list(V=1,nu=0.02)),R=list(V=1,nu=0.02))

model_FB_SSD<-MCMCglmm(SSD~FB,random=~phylo,

family="gaussian",ginverse=list(phylo=inv.phylo$Ainv),prior=prior,data=data,nitt=10000000,burnin=2500000,thin=200)

summary(model_FB_SSD)

mean(model_FB_SSD$VCV[,2])

plot(model_FB_SSD)

# MCMCglmm_FB_SGD

inv.phylo = inverseA(phylo,nodes="TIPS",scale=TRUE)

prior = list(G=list(G1=list(V=1,nu=0.02)),R=list(V=1,nu=0.02))

model_FB_SGD<-MCMCglmm(SGD~FB,random=~phylo,

family="gaussian",ginverse=list(phylo=inv.phylo$Ainv),prior=prior,data=data,nitt=10000000,burnin=2500000,thin=200)

summary(model_FB_SGD)

mean(model_FB_SGD$VCV[,2])

plot(model_FB_SGD)

# MCMCglmm_FE_open_W

inv.phylo = inverseA(phylo,nodes="TIPS",scale=TRUE)

prior = list(G=list(G1=list(V=1,nu=0.02)),R=list(V=1,nu=0.02))

model_FE_open_W<-MCMCglmm(open_W~FE,random=~phylo,

family="gaussian",ginverse=list(phylo=inv.phylo$Ainv),prior=prior,data=data,nitt=10000000,burnin=2500000,thin=200)

summary(model_FE_open_W)

mean(model_FE_open_W$VCV[,2])

plot(model_FE_open_W)

# MCMCglmm_FE_FI

inv.phylo = inverseA(phylo,nodes="TIPS",scale=TRUE)

prior = list(G=list(G1=list(V=1,nu=0.02)),R=list(V=1,nu=0.02))

model_FE_FI<-MCMCglmm(FI~FE,random=~phylo,

family="gaussian",ginverse=list(phylo=inv.phylo$Ainv),prior=prior,data=data,nitt=10000000,burnin=2500000,thin=200)

summary(model_FE_FI)

mean(model_FE_FI$VCV[,2])

plot(model_FE_FI)

# MCMCglmm_FE_MB

inv.phylo = inverseA(phylo,nodes="TIPS",scale=TRUE)

prior = list(G=list(G1=list(V=1,nu=0.02)),R=list(V=1,nu=0.02))

model_FE_MB<-MCMCglmm(MB~FE,random=~phylo,

family="gaussian",ginverse=list(phylo=inv.phylo$Ainv),prior=prior,data=data,nitt=10000000,burnin=2500000,thin=200)

summary(model_FE_MB)

mean(model_FE_MB$VCV[,2])

plot(model_FE_MB)

# MCMCglmm_FE_MNI

inv.phylo = inverseA(phylo,nodes="TIPS",scale=TRUE)

prior = list(G=list(G1=list(V=1,nu=0.02)),R=list(V=1,nu=0.02))

model_FE_MNI<-MCMCglmm(MNI~FE,random=~phylo,

family="gaussian",ginverse=list(phylo=inv.phylo$Ainv),prior=prior,data=data,nitt=10000000,burnin=2500000,thin=200)

summary(model_FE_MNI)

mean(model_FE_MNI$VCV[,2])

plot(model_FE_MNI)

# MCMCglmm_FE_MI

inv.phylo = inverseA(phylo,nodes="TIPS",scale=TRUE)

prior = list(G=list(G1=list(V=1,nu=0.02)),R=list(V=1,nu=0.02))

model_FE_MI<-MCMCglmm(MI~FE,random=~phylo,

family="gaussian",ginverse=list(phylo=inv.phylo$Ainv),prior=prior,data=data,nitt=10000000,burnin=2500000,thin=200)

summary(model_FE_MI)

mean(model_FE_MI$VCV[,2])

plot(model_FE_MI)

# MCMCglmm_FE_SSD

inv.phylo = inverseA(phylo,nodes="TIPS",scale=TRUE)

prior = list(G=list(G1=list(V=1,nu=0.02)),R=list(V=1,nu=0.02))

model_FE_SSD<-MCMCglmm(SSD~FE,random=~phylo,

family="gaussian",ginverse=list(phylo=inv.phylo$Ainv),prior=prior,data=data,nitt=10000000,burnin=2500000,thin=200)

summary(model_FE_SSD)

mean(model_FE_SSD$VCV[,2])

plot(model_FE_SSD)

# MCMCglmm_FE_SGD

inv.phylo = inverseA(phylo,nodes="TIPS",scale=TRUE)

prior = list(G=list(G1=list(V=1,nu=0.02)),R=list(V=1,nu=0.02))

model_FE_SGD<-MCMCglmm(SGD~FE,random=~phylo,

family="gaussian",ginverse=list(phylo=inv.phylo$Ainv),prior=prior,data=data,nitt=10000000,burnin=2500000,thin=200)

summary(model_FE_SGD)

mean(model_FE_SGD$VCV[,2])

plot(model_FE_SGD)

# MCMCglmm_open_W_FI

inv.phylo = inverseA(phylo,nodes="TIPS",scale=TRUE)

prior = list(G=list(G1=list(V=1,nu=0.02)),R=list(V=1,nu=0.02))

model_open_W_FI<-MCMCglmm(FI~open_W,random=~phylo,

family="gaussian",ginverse=list(phylo=inv.phylo$Ainv),prior=prior,data=data,nitt=10000000,burnin=2500000,thin=200)

summary(model_open_W_FI)

mean(model_open_W_FI$VCV[,2])

plot(model_open_W_FI)

# MCMCglmm_open_W_MB

inv.phylo = inverseA(phylo,nodes="TIPS",scale=TRUE)

prior = list(G=list(G1=list(V=1,nu=0.02)),R=list(V=1,nu=0.02))

model_open_W_MB<-MCMCglmm(MB~open_W,random=~phylo,

family="gaussian",ginverse=list(phylo=inv.phylo$Ainv),prior=prior,data=data,nitt=10000000,burnin=2500000,thin=200)

summary(model_open_W_MB)

mean(model_open_W_MB$VCV[,2])

plot(model_open_W_MB)

# MCMCglmm_open_W_MNI

inv.phylo = inverseA(phylo,nodes="TIPS",scale=TRUE)

prior = list(G=list(G1=list(V=1,nu=0.02)),R=list(V=1,nu=0.02))

model_open_W_MNI<-MCMCglmm(MNI~open_W,random=~phylo,

family="gaussian",ginverse=list(phylo=inv.phylo$Ainv),prior=prior,data=data,nitt=10000000,burnin=2500000,thin=200)

summary(model_open_W_MNI)

mean(model_open_W_MNI$VCV[,2])

plot(model_open_W_MNI)

# MCMCglmm_open_W_MI

inv.phylo = inverseA(phylo,nodes="TIPS",scale=TRUE)

prior = list(G=list(G1=list(V=1,nu=0.02)),R=list(V=1,nu=0.02))

model_open_W_MI<-MCMCglmm(MI~open_W,random=~phylo,

family="gaussian",ginverse=list(phylo=inv.phylo$Ainv),prior=prior,data=data,nitt=10000000,burnin=2500000,thin=200)

summary(model_open_W_MI)

mean(model_open_W_MI$VCV[,2])

plot(model_open_W_MI)

# MCMCglmm_open_W_SSD

inv.phylo = inverseA(phylo,nodes="TIPS",scale=TRUE)

prior = list(G=list(G1=list(V=1,nu=0.02)),R=list(V=1,nu=0.02))

model_open_W_SSD<-MCMCglmm(SSD~open_W,random=~phylo,

family="gaussian",ginverse=list(phylo=inv.phylo$Ainv),prior=prior,data=data,nitt=10000000,burnin=2500000,thin=200)

summary(model_open_W_SSD)

mean(model_open_W_SSD$VCV[,2])

plot(model_open_W_SSD)

# MCMCglmm_open_W_SGD

inv.phylo = inverseA(phylo,nodes="TIPS",scale=TRUE)

prior = list(G=list(G1=list(V=1,nu=0.02)),R=list(V=1,nu=0.02))

model_open_W_SGD<-MCMCglmm(SGD~open_W,random=~phylo,

family="gaussian",ginverse=list(phylo=inv.phylo$Ainv),prior=prior,data=data,nitt=10000000,burnin=2500000,thin=200)

summary(model_open_W_SGD)

mean(model_open_W_SGD$VCV[,2])

plot(model_open_W_SGD)

# MCMCglmm_FI_MB

inv.phylo = inverseA(phylo,nodes="TIPS",scale=TRUE)

prior = list(G=list(G1=list(V=1,nu=0.02)),R=list(V=1,nu=0.02))

model_FI_MB<-MCMCglmm(MB~FI,random=~phylo,

family="gaussian",ginverse=list(phylo=inv.phylo$Ainv),prior=prior,data=data,nitt=10000000,burnin=2500000,thin=200)

summary(model_FI_MB)

mean(model_FI_MB$VCV[,2])

plot(model_FI_MB)

# MCMCglmm_FI_MNI

inv.phylo = inverseA(phylo,nodes="TIPS",scale=TRUE)

prior = list(G=list(G1=list(V=1,nu=0.02)),R=list(V=1,nu=0.02))

model_FI_MNI<-MCMCglmm(MNI~FI,random=~phylo,

family="gaussian",ginverse=list(phylo=inv.phylo$Ainv),prior=prior,data=data,nitt=10000000,burnin=2500000,thin=200)

summary(model_FI_MNI)

mean(model_FI_MNI$VCV[,2])

plot(model_FI_MNI)

# MCMCglmm_FI_MI

inv.phylo = inverseA(phylo,nodes="TIPS",scale=TRUE)

prior = list(G=list(G1=list(V=1,nu=0.02)),R=list(V=1,nu=0.02))

model_FI_MI<-MCMCglmm(MI~FI,random=~phylo,

family="gaussian",ginverse=list(phylo=inv.phylo$Ainv),prior=prior,data=data,nitt=10000000,burnin=2500000,thin=200)

summary(model_FI_MI)

mean(model_FI_MI$VCV[,2])

plot(model_FI_MI)

# MCMCglmm_FI_SSD

inv.phylo = inverseA(phylo,nodes="TIPS",scale=TRUE)

prior = list(G=list(G1=list(V=1,nu=0.02)),R=list(V=1,nu=0.02))

model_FI_SSD<-MCMCglmm(SSD~FI,random=~phylo,

family="gaussian",ginverse=list(phylo=inv.phylo$Ainv),prior=prior,data=data,nitt=10000000,burnin=2500000,thin=200)

summary(model_FI_SSD)

mean(model_FI_SSD$VCV[,2])

plot(model_FI_SSD)

# MCMCglmm_FI_SGD

inv.phylo = inverseA(phylo,nodes="TIPS",scale=TRUE)

prior = list(G=list(G1=list(V=1,nu=0.02)),R=list(V=1,nu=0.02))

model_FI_SGD<-MCMCglmm(SGD~FI,random=~phylo,

family="gaussian",ginverse=list(phylo=inv.phylo$Ainv),prior=prior,data=data,nitt=10000000,burnin=2500000,thin=200)

summary(model_FI_SGD)

mean(model_FI_SGD$VCV[,2])

plot(model_FI_SGD)

# MCMCglmm_MB_MNI

inv.phylo = inverseA(phylo,nodes="TIPS",scale=TRUE)

prior = list(G=list(G1=list(V=1,nu=0.02)),R=list(V=1,nu=0.02))

model_MB_MNI<-MCMCglmm(MNI~MB,random=~phylo,

family="gaussian",ginverse=list(phylo=inv.phylo$Ainv),prior=prior,data=data,nitt=10000000,burnin=2500000,thin=200)

summary(model_MB_MNI)

mean(model_MB_MNI$VCV[,2])

plot(model_MB_MNI)

# MCMCglmm_MB_MI

inv.phylo = inverseA(phylo,nodes="TIPS",scale=TRUE)

prior = list(G=list(G1=list(V=1,nu=0.02)),R=list(V=1,nu=0.02))

model_MB_MI<-MCMCglmm(MI~MB,random=~phylo,

family="gaussian",ginverse=list(phylo=inv.phylo$Ainv),prior=prior,data=data,nitt=10000000,burnin=2500000,thin=200)

summary(model_MB_MI)

mean(model_MB_MI$VCV[,2])

plot(model_MB_MI)

# MCMCglmm_MB_SSD

inv.phylo = inverseA(phylo,nodes="TIPS",scale=TRUE)

prior = list(G=list(G1=list(V=1,nu=0.02)),R=list(V=1,nu=0.02))

model_MB_SSD <-MCMCglmm(SSD~MB,random=~phylo,

family="gaussian",ginverse=list(phylo=inv.phylo$Ainv),prior=prior,data=data,nitt=10000000,burnin=2500000,thin=200)

summary(model_MB_SSD)

mean(model_MB_SSD$VCV[,2])

plot(model_MB_SSD)

# MCMCglmm_MB_SGD

inv.phylo = inverseA(phylo,nodes="TIPS",scale=TRUE)

prior = list(G=list(G1=list(V=1,nu=0.02)),R=list(V=1,nu=0.02))

model_MB_SGD <-MCMCglmm(SGD~MB,random=~phylo,

family="gaussian",ginverse=list(phylo=inv.phylo$Ainv),prior=prior,data=data,nitt=10000000,burnin=2500000,thin=200)

summary(model_MB_SGD)

mean(model_MB_SGD$VCV[,2])

plot(model_MB_SGD)

# MCMCglmm_MNI_MI

inv.phylo = inverseA(phylo,nodes="TIPS",scale=TRUE)

prior = list(G=list(G1=list(V=1,nu=0.02)),R=list(V=1,nu=0.02))

model_MNI_MI<-MCMCglmm(MI~MNI,random=~phylo,

family="gaussian",ginverse=list(phylo=inv.phylo$Ainv),prior=prior,data=data,nitt=10000000,burnin=2500000,thin=200)

summary(model_MNI_MI)

mean(model_MNI_MI$VCV[,2])

plot(model_MNI_MI)

# MCMCglmm_MNI_SSD

inv.phylo = inverseA(phylo,nodes="TIPS",scale=TRUE)

prior = list(G=list(G1=list(V=1,nu=0.02)),R=list(V=1,nu=0.02))

model_MNI_SSD <-MCMCglmm(SSD~MNI,random=~phylo,

family="gaussian",ginverse=list(phylo=inv.phylo$Ainv),prior=prior,data=data,nitt=10000000,burnin=2500000,thin=200)

summary(model_MNI_SSD)

mean(model_MNI_SSD$VCV[,2])

plot(model_MNI_SSD)

# MCMCglmm_MNI_SGD

inv.phylo = inverseA(phylo,nodes="TIPS",scale=TRUE)

prior = list(G=list(G1=list(V=1,nu=0.02)),R=list(V=1,nu=0.02))

model_MNI_SGD <-MCMCglmm(SGD~MNI,random=~phylo,

family="gaussian",ginverse=list(phylo=inv.phylo$Ainv),prior=prior,data=data,nitt=10000000,burnin=2500000,thin=200)

summary(model_MNI_SGD)

mean(model_MNI_SGD$VCV[,2])

plot(model_MNI_SGD)

# MCMCglmm_MI_SSD

inv.phylo = inverseA(phylo,nodes="TIPS",scale=TRUE)

prior = list(G=list(G1=list(V=1,nu=0.02)),R=list(V=1,nu=0.02))

model_MI_SSD <-MCMCglmm(SSD~MI,random=~phylo,

family="gaussian",ginverse=list(phylo=inv.phylo$Ainv),prior=prior,data=data,nitt=10000000,burnin=2500000,thin=200)

summary(model_MI_SSD)

mean(model_MI_SSD$VCV[,2])

plot(model_MI_SSD)

# MCMCglmm_MI_SGD

inv.phylo = inverseA(phylo,nodes="TIPS",scale=TRUE)

prior = list(G=list(G1=list(V=1,nu=0.02)),R=list(V=1,nu=0.02))

model_MI_SGD <-MCMCglmm(SGD~MI,random=~phylo,

family="gaussian",ginverse=list(phylo=inv.phylo$Ainv),prior=prior,data=data,nitt=10000000,burnin=2500000,thin=200)

summary(model_MI_SGD)

mean(model_MI_SGD$VCV[,2])

plot(model_MI_SGD)

# MCMCglmm_SSD_SGD

inv.phylo = inverseA(phylo,nodes="TIPS",scale=TRUE)

prior = list(G=list(G1=list(V=1,nu=0.02)),R=list(V=1,nu=0.02))

model_SSD_SGD <-MCMCglmm(SGD~SSD,random=~phylo,

family="gaussian",ginverse=list(phylo=inv.phylo$Ainv),prior=prior,data=data,nitt=10000000,burnin=2500000,thin=200)

summary(model_SSD_SGD)

mean(model_SSD_SGD$VCV[,2])

plot(model_SSD_SGD)

itt=10000000,burnin=2500000,thin=200)

summary(model_SGD_MGC)

mean(model_SGD_MGC$VCV[,2])

plot(model_SGD_MGC)

> summary(model_FB_FE)

Iterations = 2500001:9999801

Thinning interval = 200

Sample size = 37500

DIC: 26.67239

G-structure: ~phylo

post.mean l-95% CI u-95% CI eff.samp

phylo 0.4451 0.00154 1.646 37500

R-structure: ~units

post.mean l-95% CI u-95% CI eff.samp

units 0.4767 0.002386 1.105 37500

Location effects: FE ~ FB

post.mean l-95% CI u-95% CI eff.samp pMCMC

(Intercept) -0.0259 -0.7979 0.7108 37500 0.9456

FB 0.6888 0.1377 1.2435 37500 0.0206 *

---

Signif. codes: 0 ‘***’ 0.001 ‘**’ 0.01 ‘*’ 0.05 ‘.’ 0.1 ‘ ’ 1

> mean(model_FB_FE$VCV[,2])

[1] 0.4767034

> summary(model_FB_open_W)

Iterations = 2500001:9999801

Thinning interval = 200

Sample size = 37500

DIC: -41.10291

G-structure: ~phylo

post.mean l-95% CI u-95% CI eff.samp

phylo 0.005495 0.001097 0.01256 37500

R-structure: ~units

post.mean l-95% CI u-95% CI eff.samp

units 0.003925 0.0009657 0.008439 37500

Location effects: open_W ~ FB

post.mean l-95% CI u-95% CI eff.samp pMCMC

(Intercept) 0.0425765 -0.0349953 0.1206696 37500 0.251

FB 0.0004686 -0.0570191 0.0549426 38072 0.988

> mean(model_FB_open_W$VCV[,2])

[1] 0.003925264

> plot(model_FB_open_W)

> summary(model_FB_FI)

Iterations = 2500001:9999801

Thinning interval = 200

Sample size = 37500

DIC: 20.13312

G-structure: ~phylo

post.mean l-95% CI u-95% CI eff.samp

phylo 0.9228 0.001795 2.636 37500

R-structure: ~units

post.mean l-95% CI u-95% CI eff.samp

units 0.5168 0.001586 1.454 37500

Location effects: FI ~ FB

post.mean l-95% CI u-95% CI eff.samp pMCMC

(Intercept) -0.05465 -1.07548 0.97618 36462 0.914

FB 0.45973 -0.21652 1.16095 38153 0.169

> mean(model_FB_FI$VCV[,2])

[1] 0.5167696

> summary(model_FB_MB)

Iterations = 2500001:9999801

Thinning interval = 200

Sample size = 37500

DIC: 39.07096

G-structure: ~phylo

post.mean l-95% CI u-95% CI eff.samp

phylo 0.5634 0.001422 2.663 37500

R-structure: ~units

post.mean l-95% CI u-95% CI eff.samp

units 1.027 0.002724 2.17 37500

Location effects: MB ~ FB

post.mean l-95% CI u-95% CI eff.samp pMCMC

(Intercept) -0.0317 -0.9438 0.8825 37500 0.947

FB 0.1991 -0.5123 0.9311 37500 0.548

> mean(model_FB_MB$VCV[,2])

[1] 1.027037

> summary(model_FB_MNI)

Iterations = 2500001:9999801

Thinning interval = 200

Sample size = 37500

DIC: 25.03327

G-structure: ~phylo

post.mean l-95% CI u-95% CI eff.samp

phylo 0.6145 0.001812 2.013 37500

R-structure: ~units

post.mean l-95% CI u-95% CI eff.samp

units 0.4968 0.001991 1.247 37500

Location effects: MNI ~ FB

post.mean l-95% CI u-95% CI eff.samp pMCMC

(Intercept) -0.01902 -0.90148 0.80478 37500 0.9648

FB 0.60594 -0.01896 1.20067 37500 0.0554 .

---

Signif. codes: 0 ‘***’ 0.001 ‘**’ 0.01 ‘*’ 0.05 ‘.’ 0.1 ‘ ’ 1

> mean(model_FB_MNI$VCV[,2])

[1] 0.496769

> summary(model_FB_MI)

Iterations = 2500001:9999801

Thinning interval = 200

Sample size = 37500

DIC: 22.511

G-structure: ~phylo

post.mean l-95% CI u-95% CI eff.samp

phylo 0.4642 0.001331 1.582 37500

R-structure: ~units

post.mean l-95% CI u-95% CI eff.samp

units 0.405 0.00174 0.9901 37500

Location effects: MI ~ FB

post.mean l-95% CI u-95% CI eff.samp pMCMC

(Intercept) -0.009748 -0.743340 0.741131 37623 0.9786

FB 0.723744 0.198847 1.276846 37500 0.0121 *

---

Signif. codes: 0 ‘***’ 0.001 ‘**’ 0.01 ‘*’ 0.05 ‘.’ 0.1 ‘ ’ 1

> mean(model_FB_MI$VCV[,2])

[1] 0.405031

> summary(model_FB_SSD)

Iterations = 2500001:9999801

Thinning interval = 200

Sample size = 37500

DIC: 18.71533

G-structure: ~phylo

post.mean l-95% CI u-95% CI eff.samp

phylo 0.184 0.001169 0.6761 37500

R-structure: ~units

post.mean l-95% CI u-95% CI eff.samp

units 0.2266 0.002681 0.5015 37500

Location effects: SSD ~ FB

post.mean l-95% CI u-95% CI eff.samp pMCMC

(Intercept) 0.0244 -0.4574 0.5301 38134 0.910080

FB 0.8708 0.5024 1.2423 37500 0.000213 ***

---

Signif. codes: 0 ‘***’ 0.001 ‘**’ 0.01 ‘*’ 0.05 ‘.’ 0.1 ‘ ’ 1

> mean(model_FB_SSD$VCV[,2])

[1] 0.2265945

> summary(model_FB_SGD)

Iterations = 2500001:9999801

Thinning interval = 200

Sample size = 37500

DIC: 35.51915

G-structure: ~phylo

post.mean l-95% CI u-95% CI eff.samp

phylo 0.4105 0.001706 1.869 37500

R-structure: ~units

post.mean l-95% CI u-95% CI eff.samp

units 0.7582 0.002794 1.618 37500

Location effects: SGD ~ FB

post.mean l-95% CI u-95% CI eff.samp pMCMC

(Intercept) -0.01776 -0.83575 0.70974 35630 0.9670

FB 0.55620 -0.05485 1.18431 36796 0.0707 .

---

Signif. codes: 0 ‘***’ 0.001 ‘**’ 0.01 ‘*’ 0.05 ‘.’ 0.1 ‘ ’ 1

> mean(model_FB_SGD$VCV[,2])

[1] 0.758185

> summary(model_FE_open_W)

Iterations = 2500001:9999801

Thinning interval = 200

Sample size = 37500

DIC: -40.92837

G-structure: ~phylo

post.mean l-95% CI u-95% CI eff.samp

phylo 0.005372 0.001116 0.01221 39715

R-structure: ~units

post.mean l-95% CI u-95% CI eff.samp

units 0.003944 0.00104 0.008511 37500

Location effects: open_W ~ FE

post.mean l-95% CI u-95% CI eff.samp pMCMC

(Intercept) 0.042424 -0.036319 0.118245 39213 0.248

FE 0.004543 -0.045248 0.054896 37500 0.843

> mean(model_FE_open_W$VCV[,2])

[1] 0.003944212

> summary(model_FE_FI)

Iterations = 2500001:9999801

Thinning interval = 200

Sample size = 37500

DIC: 22.94257

G-structure: ~phylo

post.mean l-95% CI u-95% CI eff.samp

phylo 0.9282 0.00174 2.748 37500

R-structure: ~units

post.mean l-95% CI u-95% CI eff.samp

units 0.5954 0.001807 1.631 37500

Location effects: FI ~ FE

post.mean l-95% CI u-95% CI eff.samp pMCMC

(Intercept) -0.04057 -1.07000 1.00805 37500 0.938

FE 0.30780 -0.30368 0.93282 37500 0.296

> mean(model_FE_FI$VCV[,2])

[1] 0.595361

> summary(model_FE_MB)

Iterations = 2500001:9999801

Thinning interval = 200

Sample size = 37500

DIC: 40.15229

G-structure: ~phylo

post.mean l-95% CI u-95% CI eff.samp

phylo 0.3765 0.001546 1.807 37500

R-structure: ~units

post.mean l-95% CI u-95% CI eff.samp

units 0.993 0.003908 2.04 37500

Location effects: MB ~ FE

post.mean l-95% CI u-95% CI eff.samp pMCMC

(Intercept) -0.01977 -0.78886 0.76403 37500 0.955

FE 0.35614 -0.26848 1.01310 37500 0.242

> mean(model_FE_MB$VCV[,2])

[1] 0.9929581

> plot(model_FE_MB)

> summary(model_FE_MNI)

Iterations = 2500001:9999801

Thinning interval = 200

Sample size = 37500

DIC: 28.50048

G-structure: ~phylo

post.mean l-95% CI u-95% CI eff.samp

phylo 0.4467 0.001657 1.715 37500

R-structure: ~units

post.mean l-95% CI u-95% CI eff.samp

units 0.5387 0.001657 1.229 37500

Location effects: MNI ~ FE

post.mean l-95% CI u-95% CI eff.samp pMCMC

(Intercept) -1.209e-07 -7.607e-01 7.622e-01 37500 0.9909

FE 6.001e-01 6.219e-02 1.136e+00 37500 0.0369 *

---

Signif. codes: 0 ‘***’ 0.001 ‘**’ 0.01 ‘*’ 0.05 ‘.’ 0.1 ‘ ’ 1

> mean(model_FE_MNI$VCV[,2])

[1] 0.5387447

> summary(model_FE_MI)

Iterations = 2500001:9999801

Thinning interval = 200

Sample size = 37500

DIC: 6.941111

G-structure: ~phylo

post.mean l-95% CI u-95% CI eff.samp

phylo 0.3899 0.002451 0.9954 37500

R-structure: ~units

post.mean l-95% CI u-95% CI eff.samp

units 0.1749 0.001977 0.5332 37500

Location effects: MI ~ FE

post.mean l-95% CI u-95% CI eff.samp pMCMC

(Intercept) 0.02304 -0.61393 0.66643 37500 0.941813

FE 0.80854 0.44089 1.17237 36747 0.000427 ***

---

Signif. codes: 0 ‘***’ 0.001 ‘**’ 0.01 ‘*’ 0.05 ‘.’ 0.1 ‘ ’ 1

> mean(model_FE_MI$VCV[,2])

[1] 0.1749049

> summary(model_FE_SSD)

Iterations = 2500001:9999801

Thinning interval = 200

Sample size = 37500

DIC: 17.33695

G-structure: ~phylo

post.mean l-95% CI u-95% CI eff.samp

phylo 0.9121 0.001685 2.404 39515

R-structure: ~units

post.mean l-95% CI u-95% CI eff.samp

units 0.4307 0.001769 1.307 38483

Location effects: SSD ~ FE

post.mean l-95% CI u-95% CI eff.samp pMCMC

(Intercept) 0.05277 -0.93016 1.06004 37500 0.912

FE 0.26946 -0.34207 0.86089 37500 0.347

> mean(model_FE_SSD$VCV[,2])

[1] 0.4307324

> summary(model_FE_SGD)

Iterations = 2500001:9999801

Thinning interval = 200

Sample size = 37500

DIC: 19.35636

G-structure: ~phylo

post.mean l-95% CI u-95% CI eff.samp

phylo 0.133 0.001493 0.5255 36558

R-structure: ~units

post.mean l-95% CI u-95% CI eff.samp

units 0.22 0.003364 0.4709 37500

Location effects: SGD ~ FE

post.mean l-95% CI u-95% CI eff.samp pMCMC

(Intercept) -0.0008783 -0.4409640 0.4204644 37500 0.995

FE 0.9215390 0.6142912 1.2506258 37500 <3e-05 ***

---

Signif. codes: 0 ‘***’ 0.001 ‘**’ 0.01 ‘*’ 0.05 ‘.’ 0.1 ‘ ’ 1

> mean(model_FE_SGD$VCV[,2])

[1] 0.2199726

> summary(model_open_W_MB)

Iterations = 2500001:9999801

Thinning interval = 200

Sample size = 37500

DIC: 40.601

G-structure: ~phylo

post.mean l-95% CI u-95% CI eff.samp

phylo 0.4919 0.001481 2.373 37500

R-structure: ~units

post.mean l-95% CI u-95% CI eff.samp

units 1.089 0.003147 2.263 38538

Location effects: MB ~ open_W

post.mean l-95% CI u-95% CI eff.samp pMCMC

(Intercept) -0.2862 -2.4449 1.9227 37500 0.783

open_W 6.2959 -40.7486 54.6981 38288 0.780

> mean(model_open_W_MB$VCV[,2])

[1] 1.08875

> summary(model_open_W_MNI)

Iterations = 2500001:9999801

Thinning interval = 200

Sample size = 37500

DIC: 18.55925

G-structure: ~phylo

post.mean l-95% CI u-95% CI eff.samp

phylo 1.128 0.001869 3.038 37500

R-structure: ~units

post.mean l-95% CI u-95% CI eff.samp

units 0.5594 0.001781 1.717 37500

Location effects: MNI ~ open_W

post.mean l-95% CI u-95% CI eff.samp pMCMC

(Intercept) -0.1282 -2.2553 1.9966 37500 0.896

open_W 2.4964 -41.0224 45.5210 38584 0.897

> mean(model_open_W_MNI$VCV[,2])

[1] 0.5594042

> summary(model_open_W_MI)

Iterations = 2500001:9999801

Thinning interval = 200

Sample size = 37500

DIC: 13.9806

G-structure: ~phylo

post.mean l-95% CI u-95% CI eff.samp

phylo 0.7485 0.001587 2.027 37500

R-structure: ~units

post.mean l-95% CI u-95% CI eff.samp

units 0.3858 0.001895 1.153 37500

Location effects: MI ~ open_W

post.mean l-95% CI u-95% CI eff.samp pMCMC

(Intercept) -1.6179 -3.3011 0.2171 37500 0.0692 .

open_W 38.0277 2.3508 73.8921 37500 0.0387 *

---

Signif. codes: 0 ‘***’ 0.001 ‘**’ 0.01 ‘*’ 0.05 ‘.’ 0.1 ‘ ’ 1

> mean(model_open_W_MI$VCV[,2])

[1] 0.3857891

> summary(model_open_W_SSD)

Iterations = 2500001:9999801

Thinning interval = 200

Sample size = 37500

DIC: 11.54072

G-structure: ~phylo

post.mean l-95% CI u-95% CI eff.samp

phylo 1.142 0.002141 2.702 37500

R-structure: ~units

post.mean l-95% CI u-95% CI eff.samp

units 0.37 0.001418 1.32 36733

Location effects: SSD ~ open_W

post.mean l-95% CI u-95% CI eff.samp pMCMC

(Intercept) 0.1891 -1.8295 2.1746 37744 0.836

open_W -3.3487 -43.4341 37.9796 37500 0.846

> mean(model_open_W_SSD$VCV[,2])

[1] 0.3699996

> summary(model_open_W_SGD)

Iterations = 2500001:9999801

Thinning interval = 200

Sample size = 37500

DIC: 30.99234

G-structure: ~phylo

post.mean l-95% CI u-95% CI eff.samp

phylo 0.7463 0.001359 2.706 37500

R-structure: ~units

post.mean l-95% CI u-95% CI eff.samp

units 0.7757 0.001809 1.854 37500

Location effects: SGD ~ open_W

post.mean l-95% CI u-95% CI eff.samp pMCMC

(Intercept) -1.1188 -3.2922 0.9036 37500 0.258

open_W 25.9330 -18.3232 69.6796 37500 0.222

> mean(model_open_W_SGD$VCV[,2])

[1] 0.7756854

> summary(model_FI_MB)

Iterations = 2500001:9999801

Thinning interval = 200

Sample size = 37500

DIC: 37.49212

G-structure: ~phylo

post.mean l-95% CI u-95% CI eff.samp

phylo 0.4466 0.001475 2.067 37500

R-structure: ~units

post.mean l-95% CI u-95% CI eff.samp

units 0.881 0.00155 1.867 37500

Location effects: MB ~ FI

post.mean l-95% CI u-95% CI eff.samp pMCMC

(Intercept) -0.01553 -0.83789 0.78619 37500 0.967

FI 0.44910 -0.16301 1.05160 36093 0.132

> mean(model_FI_MB$VCV[,2])

[1] 0.8809695

> summary(model_FI_MNI)

Iterations = 2500001:9999801

Thinning interval = 200

Sample size = 37500

DIC: 13.9098

G-structure: ~phylo

post.mean l-95% CI u-95% CI eff.samp

phylo 1.035 0.001384 2.616 35423

R-structure: ~units

post.mean l-95% CI u-95% CI eff.samp

units 0.4117 0.001428 1.354 37500

Location effects: MNI ~ FI

post.mean l-95% CI u-95% CI eff.samp pMCMC

(Intercept) 0.005692 -1.025515 1.082466 37500 0.991

FI 0.397123 -0.183033 0.971129 37500 0.161

> mean(model_FI_MNI$VCV[,2])

[1] 0.4116853

> summary(model_FI_MI)

Iterations = 2500001:9999801

Thinning interval = 200

Sample size = 37500

DIC: 27.09773

G-structure: ~phylo

post.mean l-95% CI u-95% CI eff.samp

phylo 0.6303 0.001466 2.232 37500

R-structure: ~units

post.mean l-95% CI u-95% CI eff.samp

units 0.6063 0.001718 1.468 37500

Location effects: MI ~ FI

post.mean l-95% CI u-95% CI eff.samp pMCMC

(Intercept) 0.009088 -0.860372 0.892039 36018 0.982

FI 0.432888 -0.165444 1.007600 37500 0.136

> mean(model_FI_MI$VCV[,2])

[1] 0.6063087

> summary(model_FI_SSD)

Iterations = 2500001:9999801

Thinning interval = 200

Sample size = 37500

DIC: 12.50995

G-structure: ~phylo

post.mean l-95% CI u-95% CI eff.samp

phylo 1.128 0.00203 2.714 37500

R-structure: ~units

post.mean l-95% CI u-95% CI eff.samp

units 0.3821 0.001516 1.332 37500

Location effects: SSD ~ FI

post.mean l-95% CI u-95% CI eff.samp pMCMC

(Intercept) 0.05419 -1.00756 1.15195 37500 0.913

FI 0.06427 -0.52819 0.66421 37500 0.823

> mean(model_FI_SSD$VCV[,2])

[1] 0.382071

> summary(model_FI_SGD)

Iterations = 2500001:9999801

Thinning interval = 200

Sample size = 37500

DIC: 34.98362

G-structure: ~phylo

post.mean l-95% CI u-95% CI eff.samp

phylo 0.7036 0.001279 2.807 38114

R-structure: ~units

post.mean l-95% CI u-95% CI eff.samp

units 0.8977 0.00268 2.055 36493

Location effects: SGD ~ FI

post.mean l-95% CI u-95% CI eff.samp pMCMC

(Intercept) -0.01654 -1.01983 0.91544 37500 0.975

FI 0.21172 -0.43335 0.87171 36933 0.492

> mean(model_FI_SGD$VCV[,2])

[1] 0.8977061

> summary(model_MB_MNI)

Iterations = 2500001:9999801

Thinning interval = 200

Sample size = 37500

DIC: 4.041195

G-structure: ~phylo

post.mean l-95% CI u-95% CI eff.samp

phylo 0.7468 0.002299 1.691 38802

R-structure: ~units

post.mean l-95% CI u-95% CI eff.samp

units 0.2072 0.001262 0.7947 37500

Location effects: MNI ~ MB

post.mean l-95% CI u-95% CI eff.samp pMCMC

(Intercept) 0.008832 -0.874625 0.856641 36477 0.97680

MB 0.573063 0.179176 0.976031 37500 0.00875 **

---

Signif. codes: 0 ‘***’ 0.001 ‘**’ 0.01 ‘*’ 0.05 ‘.’ 0.1 ‘ ’ 1

> mean(model_MB_MNI$VCV[,2])

[1] 0.2071562

> summary(model_MB_MI)

Iterations = 2500001:9999801

Thinning interval = 200

Sample size = 37500

DIC: 24.1081

G-structure: ~phylo

post.mean l-95% CI u-95% CI eff.samp

phylo 0.7231 0.001196 2.289 36778

R-structure: ~units

post.mean l-95% CI u-95% CI eff.samp

units 0.5771 0.001683 1.499 36726

Location effects: MI ~ MB

post.mean l-95% CI u-95% CI eff.samp pMCMC

(Intercept) 0.004466 -0.909848 0.943741 37500 0.993

MB 0.372403 -0.168018 0.904234 37500 0.150

> mean(model_MB_MI$VCV[,2])

[1] 0.5770698

Iterations = 2500001:9999801

Thinning interval = 200

Sample size = 37500

DIC: 4.130274

G-structure: ~phylo

post.mean l-95% CI u-95% CI eff.samp

phylo 1.042 0.002423 2.305 37500

R-structure: ~units

post.mean l-95% CI u-95% CI eff.samp

units 0.2385 0.001511 0.9672 38304

Location effects: SSD ~ MB

post.mean l-95% CI u-95% CI eff.samp pMCMC

(Intercept) 0.02624 -0.94601 1.06008 37500 0.954

MB -0.32452 -0.78550 0.12283 37500 0.144

> mean(model_MB_SSD$VCV[,2])

[1] 0.2385258

> summary(model_MNI_MI)

Iterations = 2500001:9999801

Thinning interval = 200

Sample size = 37500

DIC: 26.21125

G-structure: ~phylo

post.mean l-95% CI u-95% CI eff.samp

phylo 0.4633 0.001463 1.716 38564

R-structure: ~units

post.mean l-95% CI u-95% CI eff.samp

units 0.4979 0.002121 1.153 37500

Location effects: MI ~ MNI

post.mean l-95% CI u-95% CI eff.samp pMCMC

(Intercept) 0.004996 -0.763105 0.766967 37649 0.9885

MNI 0.593756 0.075753 1.094951 35534 0.0273 *

---

Signif. codes: 0 ‘***’ 0.001 ‘**’ 0.01 ‘*’ 0.05 ‘.’ 0.1 ‘ ’ 1

> mean(model_MNI_MI$VCV[,2])

[1] 0.4979116

> summary(model_MNI_SSD)

Iterations = 2500001:9999801

Thinning interval = 200

Sample size = 37500

DIC: 10.08533

G-structure: ~phylo

post.mean l-95% CI u-95% CI eff.samp

phylo 1.138 0.001484 2.714 37500

R-structure: ~units

post.mean l-95% CI u-95% CI eff.samp

units 0.3621 0.001646 1.303 31926

Location effects: SSD ~ MNI

post.mean l-95% CI u-95% CI eff.samp pMCMC

(Intercept) 0.04812 -1.04710 1.10070 37500 0.930

MNI -0.03274 -0.67196 0.64139 38109 0.899

> mean(model_MNI_SSD$VCV[,2])

[1] 0.3621168

> summary(model_MNI_SGD)

Iterations = 2500001:9999801

Thinning interval = 200

Sample size = 37500

DIC: 36.168

G-structure: ~phylo

post.mean l-95% CI u-95% CI eff.samp

phylo 0.6218 0.001493 2.659 37500

R-structure: ~units

post.mean l-95% CI u-95% CI eff.samp

units 0.9126 0.001849 2.02 38182

Location effects: SGD ~ MNI

post.mean l-95% CI u-95% CI eff.samp pMCMC

(Intercept) -0.0185 -0.9679 0.8703 37500 0.963

MNI 0.2316 -0.4551 0.8980 37500 0.460

> mean(model_MNI_SGD$VCV[,2])

[1] 0.9126326

> summary(model_MI_SSD)

Iterations = 2500001:9999801

Thinning interval = 200

Sample size = 37500

DIC: 17.39132

G-structure: ~phylo

post.mean l-95% CI u-95% CI eff.samp

phylo 0.9118 0.001763 2.414 37500

R-structure: ~units

post.mean l-95% CI u-95% CI eff.samp

units 0.4385 0.001613 1.34 37500

Location effects: SSD ~ MI

post.mean l-95% CI u-95% CI eff.samp pMCMC

(Intercept) 0.04301 -0.97538 1.01841 37500 0.931

MI 0.27223 -0.33678 0.87435 36006 0.359

> mean(model_MI_SSD$VCV[,2])

[1] 0.438502

> summary(model_MI_SGD)

Iterations = 2500001:9999801

Thinning interval = 200

Sample size = 37500

DIC: 30.29032

G-structure: ~phylo

post.mean l-95% CI u-95% CI eff.samp

phylo 0.4488 0.001618 1.748 37500

R-structure: ~units

post.mean l-95% CI u-95% CI eff.samp

units 0.5856 0.002392 1.309 37500

Location effects: SGD ~ MI

post.mean l-95% CI u-95% CI eff.samp pMCMC

(Intercept) -0.02079 -0.80495 0.73837 37500 0.9560

MI 0.65451 0.13754 1.22533 38765 0.0222 *

---

Signif. codes: 0 ‘***’ 0.001 ‘**’ 0.01 ‘*’ 0.05 ‘.’ 0.1 ‘ ’ 1

> mean(model_MI_SGD$VCV[,2])

[1] 0.585579

> summary(model_SSD_SGD)

Iterations = 2500001:9999801

Thinning interval = 200

Sample size = 37500

DIC: 35.12232

G-structure: ~phylo

post.mean l-95% CI u-95% CI eff.samp

phylo 0.5389 0.0013 2.282 37500

R-structure: ~units

post.mean l-95% CI u-95% CI eff.samp

units 0.8113 0.002775 1.793 37500

Location effects: SGD ~ SSD

post.mean l-95% CI u-95% CI eff.samp pMCMC

(Intercept) -0.03043 -0.91031 0.80956 37500 0.941

SSD 0.45127 -0.18174 1.08603 37500 0.146

> mean(model_SSD_SGD$VCV[,2])

[1] 0.8112722
